# Multi‐modal synergistic PET and MR reconstruction using mutually weighted quadratic priors

**DOI:** 10.1002/mrm.27521

**Published:** 2018-10-16

**Authors:** Abolfazl Mehranian, Martin A. Belzunce, Colm J. McGinnity, Aurelien Bustin, Claudia Prieto, Alexander Hammers, Andrew J. Reader

**Affiliations:** ^1^ Department of Biomedical Engineering, School of Biomedical Engineering and Imaging Sciences King's College London United Kingdom; ^2^ King's College London & Guy's and St Thomas' PET Centre, St Thomas' Hospital London United Kingdom

**Keywords:** Multi‐modal imaging, PET‐MRI, synergistic reconstruction

## Abstract

**Purpose:**

To propose a framework for synergistic reconstruction of PET‐MR and multi‐contrast MR data to improve the image quality obtained from noisy PET data and from undersampled MR data.

**Theory and Methods:**

Weighted quadratic priors were devised to preserve common boundaries between PET‐MR images while reducing noise, PET Gibbs ringing, and MR undersampling artifacts. These priors are iteratively reweighted using normalized multi‐modal Gaussian similarity kernels. Synergistic PET‐MR reconstructions were built on the PET maximum a posteriori expectation maximization algorithm and the MR regularized sensitivity encoding method. The proposed approach was compared to conventional methods, total variation, and prior‐image weighted quadratic regularization methods. Comparisons were performed on a simulated [^18^F]fluorodeoxyglucose‐PET and T_1_/T_2_‐weighted MR brain phantom, 2 in vivo T_1_/T_2_‐weighted MR brain datasets, and an in vivo [^18^F]fluorodeoxyglucose‐PET and fluid‐attenuated inversion recovery/T_1_‐weighted MR brain dataset.

**Results:**

Simulations showed that synergistic reconstructions achieve the lowest quantification errors for all image modalities compared to conventional, total variation, and weighted quadratic methods. Whereas total variation regularization preserved modality‐unique features, this method failed to recover PET details and was not able to reduce MR artifacts compared to our proposed method. For in vivo MR data, our method maintained similar image quality for 3× and 14× accelerated data. Reconstruction of the PET‐MR dataset also demonstrated improved performance of our method compared to the conventional independent methods in terms of reduced Gibbs and undersampling artifacts.

**Conclusion:**

The proposed methodology offers a robust multi‐modal synergistic image reconstruction framework that can be readily built on existing established algorithms.

## 1 INTRODUCTION

PET and MRI systems have opened the way for synergistic reconstruction of PET‐MR data to improve image quality,^1^, ^2^, ^3^ particularly for low‐count PET data and/or highly undersampled MRI data.

It is known that PET reconstruction using the conventional maximum‐likelihood expectation maximization (MLEM) algorithm exhibits noise and loss of details due to noise in the data and the limited detector resolution. Hence, coregistered high‐resolution MR images have been used to guide the reconstruction of PET data using maximum a posteriori (MAP) expectation maximization (MAPEM) algorithms. Quadratic (Tikhonov) and total variation (TV) priors are among the most commonly used MAP priors.^4^, ^5^ However, mismatches between PET and MR images may introduce false features or suppress true ones in the reconstructed images. Nonetheless, the complementary information of multi‐contrast MR images available in simultaneous PET‐MR scanners can be employed to cope with these mismatches.^6^

On the other hand, MR imaging often requires long acquisitions, particularly for multi‐parametric imaging. Conventional fast acquisitions include partial Fourier^7^ and parallel MRI such as sensitivity encoding,^8^ in which the acquisition is accelerated by undersampling the k‐space data. At high acceleration factors, the conventional reconstructions show extensive noise amplification and/or aliasing artifacts. Hence, similar to PET, different regularization methods have been investigated for incorporation of prior knowledge into MR image reconstruction,^9^, ^10^ among which compressed sensing and sparsity regularization are the most successful ones.^11^, ^12^, ^13^ In multi‐contrast and longitudinal MR scans, existing MR images of the same or different contrasts can be also used to form additional prior knowledge about the MR image being reconstructed.^14^, ^15^, ^16^, ^17^, ^18^, ^19^ Similar to MR‐guided PET reconstruction, prior image‐guided MR reconstruction is also subject to the mismatches between MR images, hence the joint or synergistic reconstruction of multi‐contrast undersampled MR images have been explored.^20^, ^21^, ^22^ Bilgic et al.^23^ proposed reconstruction of MR images using joint image gradients of multi‐contrast images. Weizman et al.^21^ studied separate TV priors defined on each MR image contrast and an additional reweighted L_1_ norm prior defined on the difference of the MR images.

Synergistic PET and MR image reconstruction has also been recently explored to exploit the complementary information of the PET‐MR images. The benefits of such reconstructions are challenged by the need for the development of 1) a model‐based joint prior that favors common features between PET and multi‐contrast MR images, irrespective of their relative signal intensities and their relative contrast orientations, while preserving modality unique features; and 2) a robust and stable optimization algorithm with preferably few hyperparameters. Ehrhardt et al.^1^ reported the first attempt in synergistic PET‐MR image reconstruction based on the parallelism of PET‐MR level sets, whereas Knoll et al.^2^ proposed a total generalized variation (TGV) regularization based on the nuclear norm. Despite promising results, their methods depend on relative signal intensities. In Ref. 3, we recently proposed a generalized TV prior with an alternating scaling scheme to handle the relative signal intensity issue. Simulation results showed that our algorithm can outperform the PET‐MR level sets and joint TV priors; however, the proposed scaling scheme was designed to match the magnitude of PET and MR image gradients using a single global scale factor. Hence, this algorithm is not efficient and robust for all regions in PET‐MR images with different gradient magnitudes. In addition, as in previous work, a relatively complex optimization algorithm was chosen.

In this study, we propose a framework for synergistic PET and multi‐contrast MR image reconstruction. In this framework, the PET and MR images are reconstructed using well‐established EM and iterative SENSE reconstruction algorithms and are regularized using adaptively weighted multi‐modal quadratic priors. These priors (1) are able to preserve modality unique features through calculating weighting factors from all image modalities, (2) are independent of the *relative* signal intensities and contrast orientations of MR or PET‐MR images, and (3) easily accommodate synergistic reconstruction of multiple PET or MR datasets. Synergistic reconstruction of multiple datasets has also been recently reported in Ref. 24. The proposed prior is similar to the Bowsher prior but different in that similarity coefficients are progressively derived from all multi‐modal images rather than being precalculated.^6^ In this study, we present our results using realistic 3D simulations, in vivo undersampled 3D MR data, and an in vivo PET‐MR dataset for the different guided and synergistic reconstruction methods.

## 2 THEORY

### 2.1 Synergistic reconstruction of PET and MR data

The synergistic reconstruction of the PET image, $u\in R^{N_{u}}$ and MR images, ${v^{\left(k\right)}}^{\in }C^{N_{k}}$, k=1,⋯,V, of different contrasts can be achieved by the following optimization^3^:

(1) $$\left(\hat{u},{\hat{v}}^{\left(1\right)},\cdots ,{\hat{v}}^{\left(V\right)}\right)=\underset{u,v^{\left(1\right)},\cdots ,v^{\left(V\right)}}{\text{argmax}}\left\{\left(\sum_{i=1}^{M_{u}}\left({y_{i}\text{log}\left({\left[Pu\right]}_{i}+{\overset{-}{r}}_{i}\right)-\left[Pu\right]}_{i}-{\overset{-}{r}}_{i}\right)\right)-\left(\frac{1}{2}\sum_{k=1}^{V}\sum_{l=1}^{L}\sum_{i=1}^{M_{k}}{w_{\mathrm{li}}^{\left(k\right)}\left|{\left[E^{\left(k\right)}v^{\left(k\right)}\right]}_{\mathrm{li}}-s_{\mathrm{li}}^{\left(k\right)}\right|}^{2}\right)-R\left(u,v^{\left(1\right)},\cdots ,v^{\left(V\right)}\right)\right\}$$

where the 3 terms of the objective function correspond to the PET data fidelity, MR data fidelity, and joint modality prior. $y\in Z^{M_{u}}$ is the PET sinogram data; $P\in R^{{M_{u}\times N}_{u}}$ is the PET system matrix (composed of the geometric transition matrix, the scanner's point spread function, and attenuation and normalization factors); $\overset{-}{r}\in R^{M_{u}}$ is an estimate of the mean PET background coincidences (randoms and scatters); and $N_{u}$ and $M_{u}$ are the number of image voxels and sinogram bins. $s^{\left(k\right)}\in C^{M_{k}L}$ is the k‐space data for the *k*th MR image contrast; $E^{\left(k\right)}\in C^{M_{k}L\times N_{k}}$is its corresponding MR encoding matrix (composed of a discrete Fourier transform, k‐space undersampling matrix, and coil sensitivity profiles). $M_{k}$, L, and $N_{k}$are the number of k‐space samples, coils, and voxels for the *k*th image contrast, respectively. $w_{\mathrm{li}}^{\left(k\right)}$ is an element of a $W^{\left(k\right)}\in R^{M_{k}L\times M_{k}L}$ weighting matrix obtained from the inversion of the noise covariance matrix.^25^ In this study, the joint prior R was defined as the sum of mutually weighted quadratic priors as follows:

(2) $$R\left(u,v^{\left(1\right)},\cdots ,v^{\left(V\right)}\right)=\frac{1}{2}\sum_{j=1}^{N_{u}}\sum_{b\in N_{j}}{\beta_{u}\xi }_{\mathrm{jb}}^{u}\omega_{\mathrm{jb}}^{u}{\left(u_{j}-u_{b}\right)}^{2}+\frac{1}{2}\sum_{k=1}^{V}\sum_{j=1}^{N_{k}}\sum_{b\in N_{j}}\beta_{v^{\left(k\right)}}\xi_{\mathrm{jb}}^{v^{\left(k\right)}}\omega_{\mathrm{jb}}^{v^{\left(k\right)}}{\left(v_{j}^{\left(k\right)}-v_{b}^{\left(k\right)}\right)}^{2}$$

where β is a regularization parameter, and $\xi_{\mathrm{jb}}$and $\omega_{\mathrm{jb}}$ are coefficients used to modulate the intensity differences between voxel j and b based on their Euclidean proximity and intensity similarity in a neighborhood $N_{j}$, respectively. The proximity coefficients were defined as:

(3) $$\xi_{\mathrm{jb}}=\frac{1}{\sqrt{\sum_{i=1}^{3}{\left(j^{\left(i\right)}-b^{\left(i\right)}\right)}^{2}}},$$

where $\left\{j^{\left(i\right)}\right\}$ and $\left\{b^{\left(i\right)}\right\}$ are the Cartesian coordinates of the jth and bth voxel. In the proposed prior, the similarity coefficients are alternatingly calculated from both PET‐MR images using the following joint coefficients (6):

$$\omega_{\mathrm{jb}}=\frac{G\left({\hat{u}}_{j},{\hat{u}}_{b},\sigma_{u}\right)G\left({\hat{v}}_{j}^{\left(1\right)},{\hat{v}}_{b}^{\left(1\right)},\sigma_{1}\right)\cdots G\left({\hat{v}}_{j}^{\left(V\right)},{\hat{v}}_{b}^{\left(V\right)},\sigma_{v^{\left(V\right)}}\right)}{\sum_{j=1}^{N}G\left({\hat{u}}_{j},{\hat{u}}_{b},\sigma_{u}\right)G\left({\hat{v}}_{j}^{\left(1\right)},{\hat{v}}_{b}^{\left(1\right)},\sigma_{v^{\left(1\right)}}\right)\cdots G\left({\hat{v}}_{j}^{\left(V\right)},{\hat{v}}_{b}^{\left(V\right)},\sigma_{v^{\left(V\right)}}\right)}$$

(4) $$G\left(q,r,\sigma \right)=\frac{1}{\sqrt{2\pi }\sigma }\text{exp}\left(-\frac{{\left(q-r\right)}^{2}}{2\sigma^{2}}\right),$$

where $\hat{u}$ and ${\hat{v}}^{\left(k\right)}$ are the current estimates of the PET and MR images, obtained iteratively in the case of synergistic reconstruction, or are prior images in the case of guided PET or MR reconstruction. These coefficients are composed of the product of Gaussian similarity kernels calculated between voxel j and b in a neighbourhood $N_{j}$ for each image modality. The role of $\omega_{\mathrm{jb}}$ is to assign a lower penalty on the local differences that are associated with a boundary identified uniquely from the PET image or MR image or mutually from all PET and MR images. In PET unique boundaries, the MR‐derived Gaussian kernels in $\omega_{\mathrm{jb}}$ are uniform, whereas in shared boundaries they have the same structural similarity irrespective of contrast orientation and relative signal intensity. Therefore, the product of the kernels will preserve the modality unique boundaries and encourage the formation of shared ones.

Because PET and MR images may all have different matrix and voxel sizes, the $\omega_{\mathrm{jb}}$ coefficients in Equation [Link] must be uniquely calculated for each modality. Hence, registration and resampling operators, $\Phi_{x\to y}$, need to be defined to spatially map image modality, x, to a given image, y (see step 3 in the proposed algorithm for more details). In this study, we followed an alternating optimization of Equation (1). As summarized in the proposed algorithm*, *the optimization consists of 3 main steps: (1) MAPEM image reconstruction of PET data using a weighted quadratic prior, employing De Pierro's decoupling rule for regularization^26^, ^27^ with $P_{\mathrm{iter}}$ iterations; (2) SENSE MR image reconstruction using a weighted quadratic prior and the conjugate gradient (CG) algorithm^28^ with $M_{\mathrm{iter}}$ iterations; and (3) calculation of the similarity coefficients used during PET and MR reconstruction.

In this algorithm, q is PET sensitivity image; D is a derivative matrix for calculation of local differences between image voxels; and ξ and ω are diagonal weighting matrices with diagonal elements calculated by Equations (3) through [Link], respectively. In this study, the proposed synergistic algorithm was employed for different synergistic PET‐MR and MR reconstructions in comparison with a number of separate reconstruction methods, as summarized in Supporting Information Table S1.

2.2

Proposed algorithm: synergistic reconstruction of PET and MR data


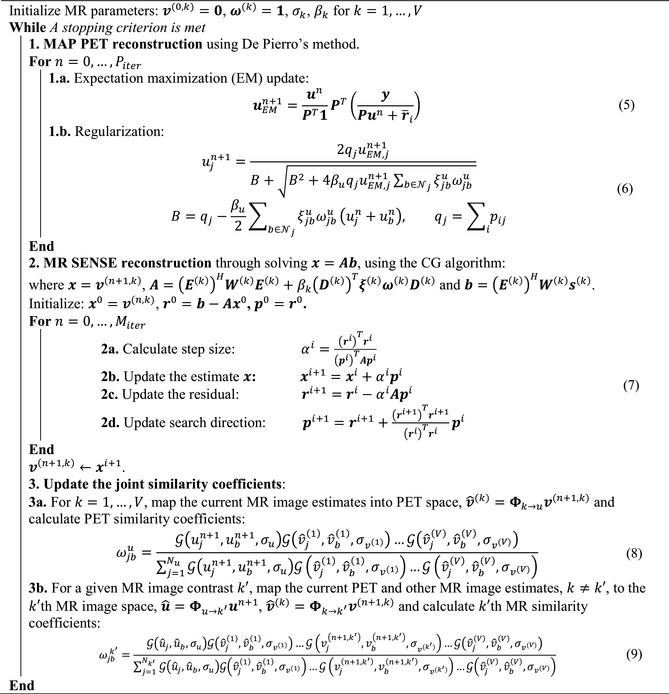


## 3 METHODS

### 3.1 Simulated and real datasets

#### 3.1.1 PET‐MR simulation

**Figure 1:**
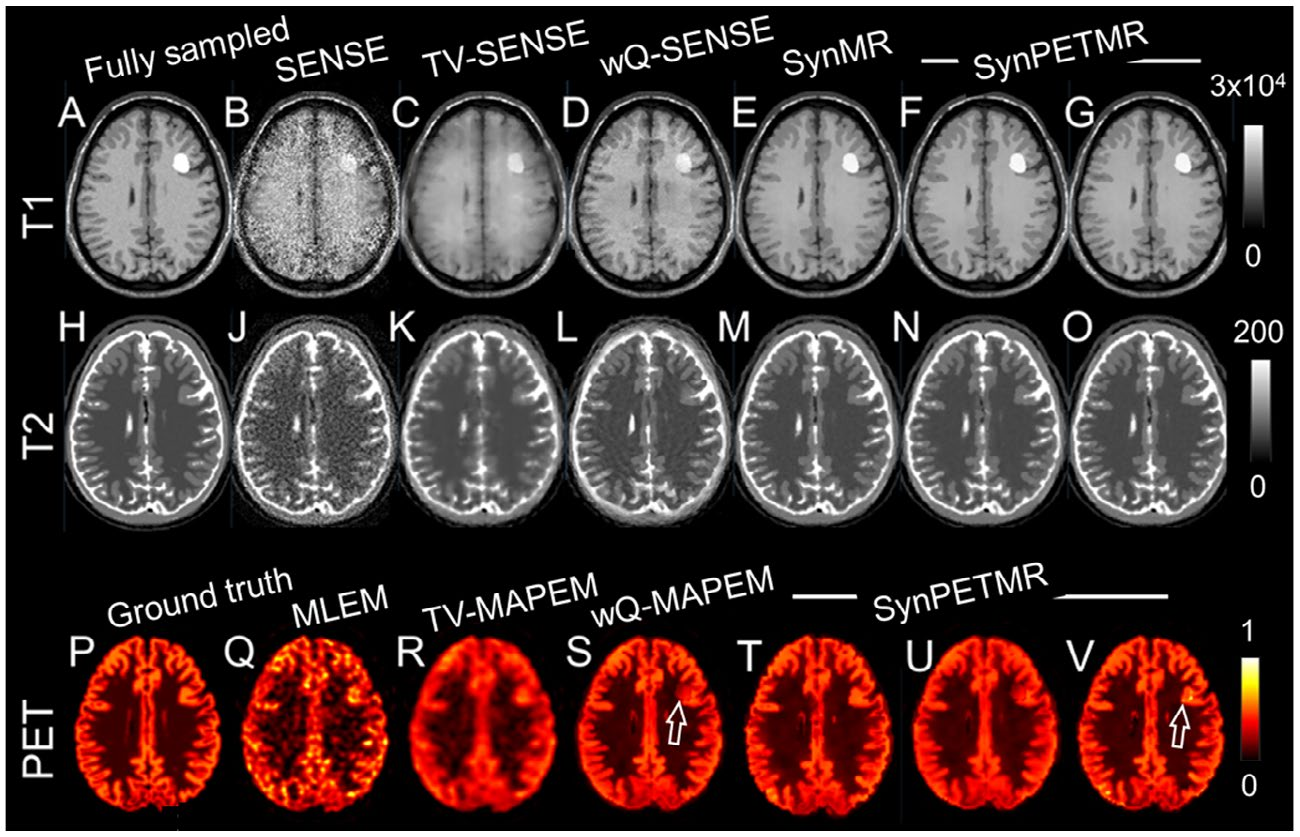
Reconstruction results for the simulated T_1_, T_2_, and PET data showing T_1_ unique lesion. Captions categorize the reconstructions in different groups. (A,H) SENSE reconstruction of fully sampled data; (C,J) SENSE reconstruction of undersampled data; (C,K) TV‐SENSE reconstruction of undersampled data; (D,L) wQ‐SENSE reconstruction of undersampled T_1_ and T_2_ data weighted using fully sampled T_2_ and T_1_ images, respectively; (E,M) synergistic reconstruction of undersampled T_1_ and T_2_ data; (F,U) synergistic reconstruction of undersampled T_1_ and PET data; (N,T) synergistic reconstruction of undersampled T2 and PET data; and (G,O,V) synergistic reconstruction of undersampled T_1_, T_2_, and PET data. (P) PET ground truth, (Q) MLEM, (R) TV‐MAPEM, and (S) wQ‐MAPEM weighted using fully sampled T_1_ image. Note that the PET images have been resampled to T_1_ MR resolution. MLEM, maximum‐likelihood expectation maximization; TV‐MAPEM, total variation‐maximum a posteriori (MAP) expectation maximization; wQ, weighted quadratic.

**Figure 2:**
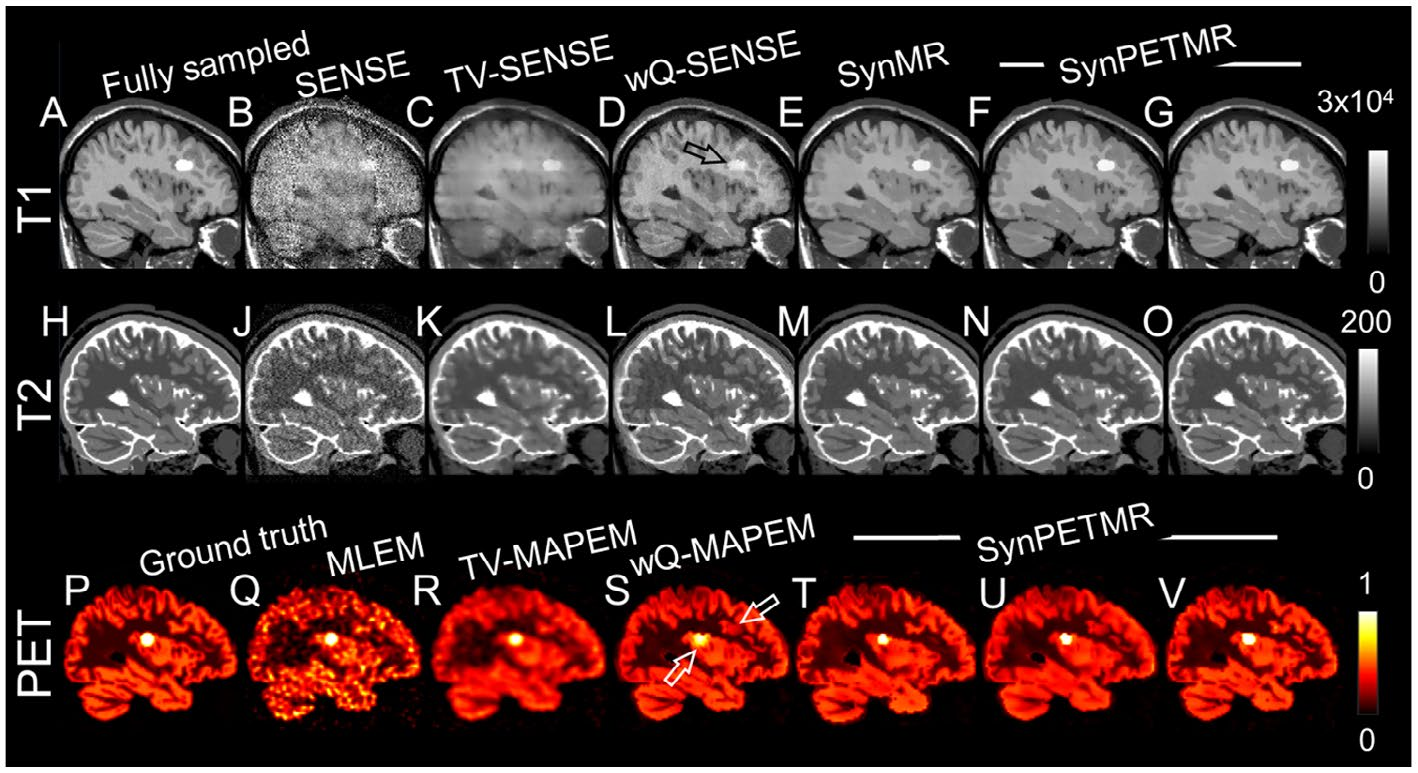
Same as Figure 1, but for a sagittal slice showing T_1_ and PET unique lesions.

The BrainWeb phantom^29^ was used to simulate an activity distribution of [^18^F]fluorodeoxyglucose (FDG) in the brain, along with T_1_‐ and T_2_‐weighted MR images. The matrix and voxel sizes of the PET phantom were set to 344 × 344 × 127 and 2.086 × 2.086 × 2.03 mm^3^, whereas those of the MR phantoms were set to 230 × 230 × 254 and 1.043 × 1.043 × 1.015 mm^3^. For the PET image, a gray‐to‐white matter activity ratio of 4:1 was considered, whereas the MR intensity ratios were obtained from the BrainWeb simulator. Unique lesions were introduced in the PET and T_1_‐weighted MR images with a volume of 0.76 mL (5.6‐mm diameter) and 0.87 mL (7.6‐mm diameter), respectively (see Figures 1–2 in the Results section for the location of the lesions). For the FDG‐PET and T_1_‐MR images, the lesion‐to‐white matter activity/intensity ratios were set to 6:1 and 2:1, respectively. PET simulations were performed for the Siemens Biograph mMR scanner (Siemens Healthcare), including attenuation, normalization factors, 10% randoms, and 30% scatter coincidences. Poisson noise realizations with a total of 90 million counts were generated for the PET phantom. Resolution degradation was modeled in image space with a 4.5‐mm Gaussian kernel.^30^ MR simulations were performed for an MR scanner with a 5‐channel coil, 100‐mm coil radius, and 150‐mm coil distance from the center. Coil sensitivity maps were simulated based on the Biot–Savart law.^31^ For undersampled MR reconstructions, the k‐spaces of the T_1_ and T_2_ images were contaminated with Gaussian noise and undersampled using Cartesian (undersampling factor of 6) and radial trajectories (20 radial spokes, undersampling factor of ~10), respectively. Supporting Information Table S2 summarizes the MR undersampling used in all experiments in this study.

#### 3.1.2 MRI in vivo dataset

Two healthy volunteers underwent undersampled T_1_‐ and T_2_‐weighted 3D whole brain MR scans on a 1.5T Siemens MR scanner (Siemens Healthcare) using a prototype variable density Cartesian acquisition with spiral profile order (VD‐CASPER) with undersampling factors (R) of 3, 9, and 14 (see Ref. 32 for more detail on the sampling). The k‐space data were acquired using a 16‐channel head coil. T_1_ images were acquired using 3D T_1_‐MPRAGE with the following parameters: TR: 1700 ms, TE: 2.5 ms, TI: 900 ms, echo spacing: 6.24 ms, flip angle: 9 degrees. Acquisition were performed fully sampled and with R = 3, 9, 14, resulting in acquisition times of 377 s, 125 s, and 83 s, respectively. T_2_ images were acquired using a 3D balanced SSFP sequence with the following parameters: TR: 5000 ms, TE: 2.57 ms, flip angle: 9 degrees. The voxel size of the T_1_ and T_2_ images of the first volunteer was set to 1.4 × 1.4 × 1.4 mm^3^, whereas for the second volunteer it was set to 1.2 × 1.2 × 1.2 mm^3^.

#### 3.1.3 PET‐MRI in vivo dataset

A patient with dementia underwent a brain PET‐MR scan on the Siemens mMR scanner and the following datasets were acquired: (1) a 30‐min PET scan with an injected activity of 212.82 MBq of [^18^F]FDG; (2) a Dixon and a UTE MR sequence to generate a 4‐tissue class (air, soft tissue, fat, and bone) attenuation map for PET attenuation correction; (3) an MPRAGE sequence with the following parameters: 5 channels, TR: 1700 ms, TE: 2.63 ms, TI: 900 ms, echo spacing: 6.24 ms, flip angle: 9 degrees, acquisition times: 142 s; and (4) a 2×‐accelerated fluid‐attenuated inversion recovery (FLAIR) MR sequence with the following parameters: 14 channels, TR: 5000 ms, TE: 395 ms, T_1_: 1800 ms, echo spacing: 6.24 ms, flip angle: 120 degrees, acquisition times: 397 s. For PET reconstruction, all correction sinograms were generated using e7 tools (Siemens offline reconstruction software; Siemens Healthcare), and images were reconstructed with PSF modeling using 4.5‐mm Gaussian kernels^30^ and the scanner's default matrix size, as used in our simulations. The k‐space of the T_1_ dataset was retrospectively undersampled using Cartesian trajectories in the phase‐ and slice‐encoding directions, each with a factor of 3, leading to a total acceleration factor R = 9. The k‐space of the FLAIR dataset was further retrospectively undersampled in the slice‐encoding direction by a factor of 3, leading to R = 6. The T_1_ and FLAIR images were reconstructed in their native matrix and voxel sizes of 512 × 244 × 244, 1.05 × 1.05 × 1.1 mm^3^; and 512 × 256 × 160, 0.48 × 0.48 × 1.0 mm^3^, respectively. Supporting Information Table S2 summarizes this experiment.

### 3.2 Reconstruction methods

The images of both the simulation and in vivo data were reconstructed as complex‐valued; however, the images presented in the Results section are magnitude images. Coil sensitivity maps were calculated by dividing the MR image from each coil by the square root of the sum of squares of all the images obtained from all the coils. In this study, the neighborhood size, $N_{j}$, of the quadratic priors in Equation (2) was set to 5 × 5 × 5 for the simulations, whereas for the in vivo datasets it was set to 3 × 3 × 3 to reduce the computational burden of our reconstructions. The σ and β parameters were set experimentally for all reconstruction setups. To facilitate and standardize the selection of σ for different images and different datasets, we normalized each image to [0,1] prior to calculation of the Gaussian kernels. Supporting Information Table S3 provides all parameters chosen for the reconstruction of the simulated and real datasets. The PET forward and back projections were implemented in C++ with GPU acceleration. MR reconstructions were performed in MatLab (MathWorks, Inc., Natick, MA).

### 3.3 Evaluation metrics

For the simulations, the performance of different reconstruction methods was quantitatively evaluated compared to a reference image (for PET, the ground truth; for MR, a reconstruction from fully sampled data) using (1) a voxel‐level error, defined in Equation (4); (2) a region‐level error, calculated from the mean of the voxel‐level errors in a region of interest (ROI); and (3) the contrast‐to‐noise ratio (CNR) for lesions, defined in Equation (5),

(5) $$\text{Error}=100\times \frac{\left|x_{j}\right|-\left|x_{j}^{\mathrm{ref}}\right|}{\left|x_{j}^{\mathrm{ref}}\right|}$$

(6) $$CNR=20{\text{log}}_{10}\left(\frac{{\overset{-}{A}}_{L}-{\overset{-}{A}}_{B}}{{\bar{\mathrm{SD}}}_{B}}\right),$$

where $x_{j}$ and $x_{j}^{\mathrm{ref}}$ are the *j*th voxel of a given (complex) image and its corresponding reference image, respectively. ${\overset{-}{A}}_{L}$ and ${\overset{-}{A}}_{B}$ are the means of the PET activity or MRI intensity in a given lesion and a background region, and ${\bar{\mathrm{SD}}}_{B}$ is the mean SD of activity/intensity in a background region. The background region was defined using 15 ROIs (~10‐mm diameter) in different regions of the brain (as shown in Supporting Information Figure S1). ROIs of the lesions were defined by thresholding the simulated PET‐MR images at a threshold of 60% of the maximum lesion value. The mean (M) and SD of voxel‐level errors in gray and white matter of each reconstruction were summarized by a root sum of squared (RSS) errors as follows:

(7) $$\text{RSS}=\sqrt{M^{2}+SD^{2}}.$$

For in vivo data, the CNR between the gray and white matter was calculated to evaluate the performance of the algorithms in the absence of a reference image.

## 4 RESULTS

### 4.1 Simulations

In Figures 1 and 2, the reconstruction results of the simulated brain phantom are shown. For visualization, PET images were resampled into MR resolution. Figures 1A through 1G show (A) fully sampled SENSE; (B) undersampled SENSE; (C) undersampled SENSE with TV regularization; (D) undersampled SENSE with quadratic regularization weighted using fully sampled T_2_ image; (E) synergistic reconstruction of undersampled T_1_ and T_2_ images; (F) synergistic reconstruction of undersampled T_1_ and PET; and (G) synergistic reconstruction of undersampled T_1_, T_2_, and PET images. Figures 1H through 1O show the same results for the T_2_ image.

Figures 1P through 1V shows (P) PET ground truth; (Q) MLEM reconstruction; (R) MAPEM reconstruction with TV regularization; (S) MAPEM with quadratic regularization weighted using fully sampled T_1_ image; (T) synergistic reconstruction of undersampled T2 and PET images; (U) synergistic reconstruction of undersampled T_1_ and PET images; and (V) synergistic reconstruction of undersampled T_1_, T_2_, and PET images. Figure 2 show the same results for a sagittal slice containing mismatched PET and T_1_ lesions. Supporting Information Figures S2 through S5 show the same results with error maps and zoomed‐in subfigures over mismatches.

The results show that SENSE reconstructions lead to noisy estimates, particularly for the T_1_ image with 6‐fold Cartesian undersampling. TV‐SENSE reconstructions reduce noise and aliasing artifacts specifically in the T_2_ image for which the radial undersampling results in incoherent artifacts suitable for sparsity regularization. However, the reconstructions do not recover all the details compared to wQ‐SENSE, which is guided by an artifact‐free MR image. In wQ‐SENSE reconstructions, there are some residual folding artifacts and suppressed/deformed edges, as shown in the zoomed‐in subfigures.

The PET reconstruction results in Figures 1 and 2 show that the MLEM reconstructions suffer from noise and loss of details, whereas TV‐MAPEM notably reduces the noise but induces blurring. The wQ‐MAPEM method improves recovery of boundaries but at the cost of inducing tumor‐like artifacts for the MR unique lesion and suppressing and deforming the PET unique lesion (see also Supporting Information Figures S2 and S5). The synergistic reconstruction of the PET and T_1_ images induces similar artifacts in the PET image, as shown in Figures 1 through 2 (S). The synergistic reconstruction of all datasets together can mitigate these artifacts; however, as shown in Figure 1V, this algorithm has introduced a false edge through preserving noise at the edge that corresponds to the MR unique lesion. Despite this, the proposed method can preserve unique lesions. However, the results in Figures 2T through 2V show that the PET lesion's size has slightly shrunk by this method compared to the MLEM and TV‐MAPEM methods.

**Figure 3:**
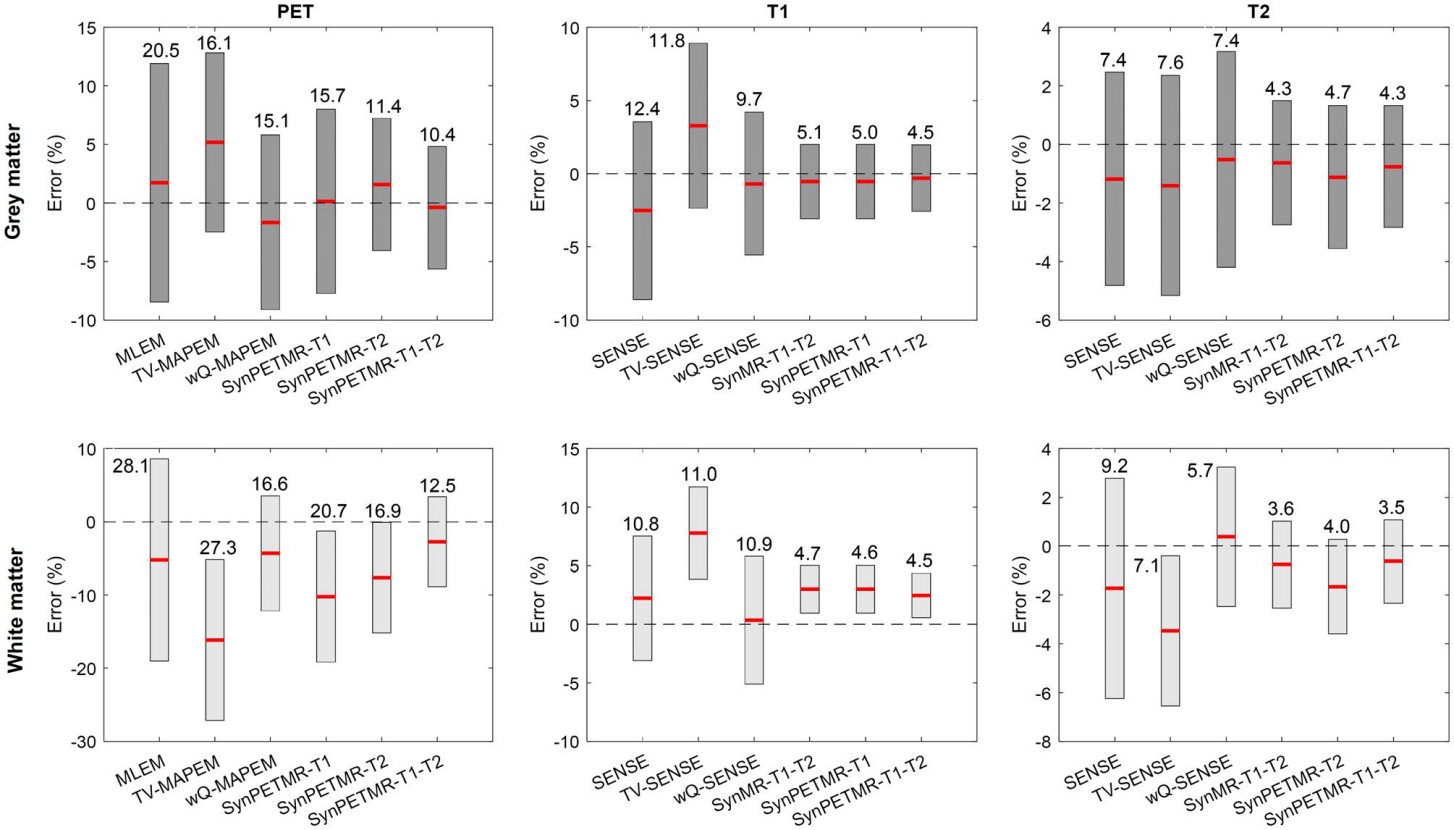
Mean (horizontal bold lines) and SD (vertical bars) of voxel‐wise errors in gray and white matter for different reconstruction methods together with their root sum of squared errors (numbers shown above each bar).

Figure 3 compares the reconstruction methods in terms of mean and SD of voxel‐level errors in gray and white matter for the PET, T_1_, and T_2_ images. The numbers above each bar in the figure report RSS errors, as defined in Equation 13. The results show that by moving from conventional reconstruction methods to synergistic ones, the mean and SD of the errors are reduced in both the gray and white matter. The conventional MLEM and SENSE methods result in an average RSS error of 13.4% in the gray matter and 16.0% in the white matter, whereas the proposed synergistic reconstructions of PET and T_1_ and T_2_ MR (SynPETMR‐T_1_‐T_2_) methods reduce these errors by more than half. The results show that PET and MR reconstructions using weighted quadratic regularization achieve a better performance than those using TV regularization; however, they are outperformed by the synergistic methods.

**Figure 4:**
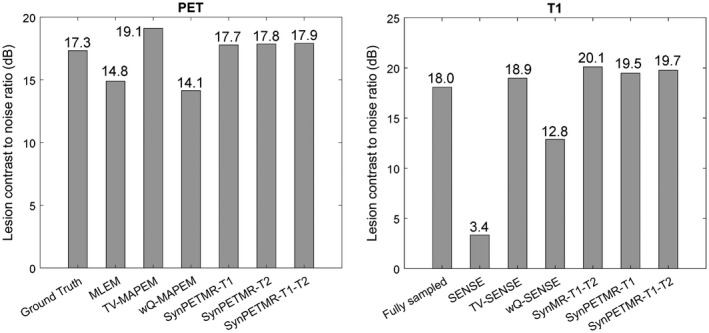
CNR results for the separate and synergistic MR and PET‐MR reconstructions. CNR, contrast‐to‐noise ratio.

Figure 4 shows CNR performance of the reconstructions for PET and T_1_ lesions. As shown, the MLEM and SENSE reconstructions result in low CNRs because these methods increase background noise, whereas the TV‐MAPEM and TV‐SENSE methods result in relatively high CNRs because the edge‐preserving TV regularization suppresses background noise and increases lesion contrast. The wQ‐MAPEM and wQ‐SENSE methods achieve lower CNR compared to their TV counterparts because they tend to suppress unique lesions due to mismatches between reconstructed images and their prior images used for guided reconstructions. Synergistic reconstruction results in a comparable and high CNR because they tend to reduce noise and preserve modality‐unique lesions.

For simulations, the reconstruction methods were performed with a large number of updates (up to 1200 updates) to ensure their convergence, as summarized in Supporting Information Table S3. In Supporting Information Figure S7, the convergence of the reconstructions is shown in terms of normalized root mean square error in the whole brain. As expected, MLEM and undersampled SENSE reconstructions give rise to noisy solutions at high iteration numbers; therefore, earlier termination is required. The results show that most of the algorithms have converged to a fixed‐point solution, particularly for the PET and T_2_ images. Moreover, the synergistically reconstructed data result in the lowest normalized root mean square error in the whole brain.

### 4.2 In vivo MRI data

**Figure 5:**
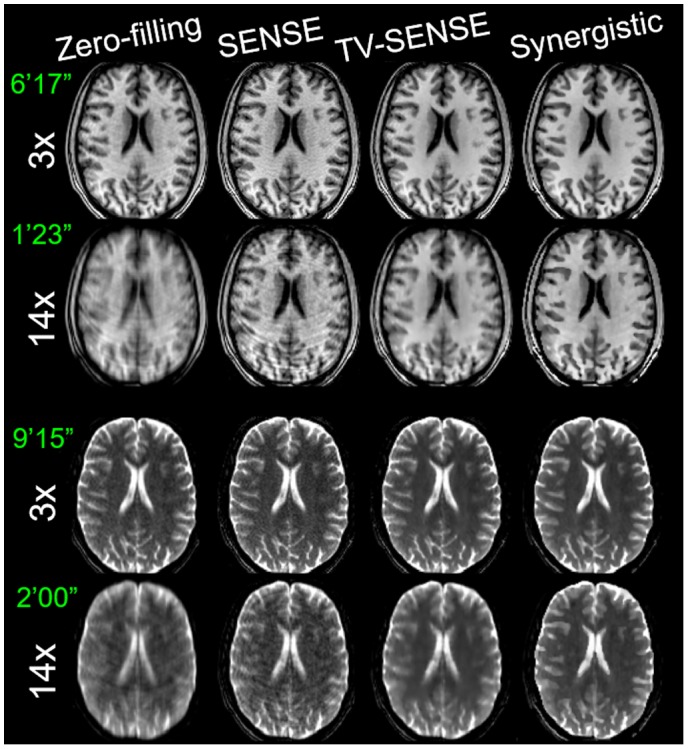
Synergistic reconstruction of the prospectively undersampled T_1_ (left) and T_2_ (right) datasets for a healthy volunteer. Acceleration factor and resulting acquisition time (in minutes and seconds) of each scan are shown.

Figures 5 through 6 compare the synergistic image reconstruction of 2 in vivo T_1_/T_2_ datasets with conventional zero‐filling, SENSE, and separate TV‐SENSE reconstruction methods for acceleration factors of 3 and 14. Supporting Information Figures S7 and S8 compare the results for all acceleration factors (including 9×) for sagittal slices. Zero‐filling images were obtained by filling the unmeasured k‐space data with zeros and were reconstructed using the conventional sum‐of‐squares method. Note that in the absence of a fully sampled MR dataset, the wQ‐SENSE method was not considered in this experiment. In the VD‐CASPER sequence used for this dataset, the sampling of k‐space is reduced from the center toward the periphery of k‐space in a spiral and random fashion. Hence, at higher accelerations the reconstructed images suffered from blurring as well as aliasing artifacts. At 3× acceleration, the TV‐SENSE and synergistic (i.e., SynMR) methods show a fairly similar performance, whereas the zero‐filling and SENSE reconstructions show blurring and noise. At higher acceleration, the TV‐SENSE reconstructions show blurring and residual artifacts, whereas the synergistic method tends to keep the image quality comparable to the 3×‐accelerated images for both the T_1_ and T_2_ datasets. The arrows point to the regions with notable differences between the reconstruction methods. Supporting Information Figure S9 compares the CNR performance of the reconstructions between gray and white matter in T_1_ and T_2_ images for different acceleration factors. As shown, at each acceleration factor the proposed method achieves the highest CNR thanks to a higher contrast between the gray and white matter and lower background variation.

**Figure 6:**
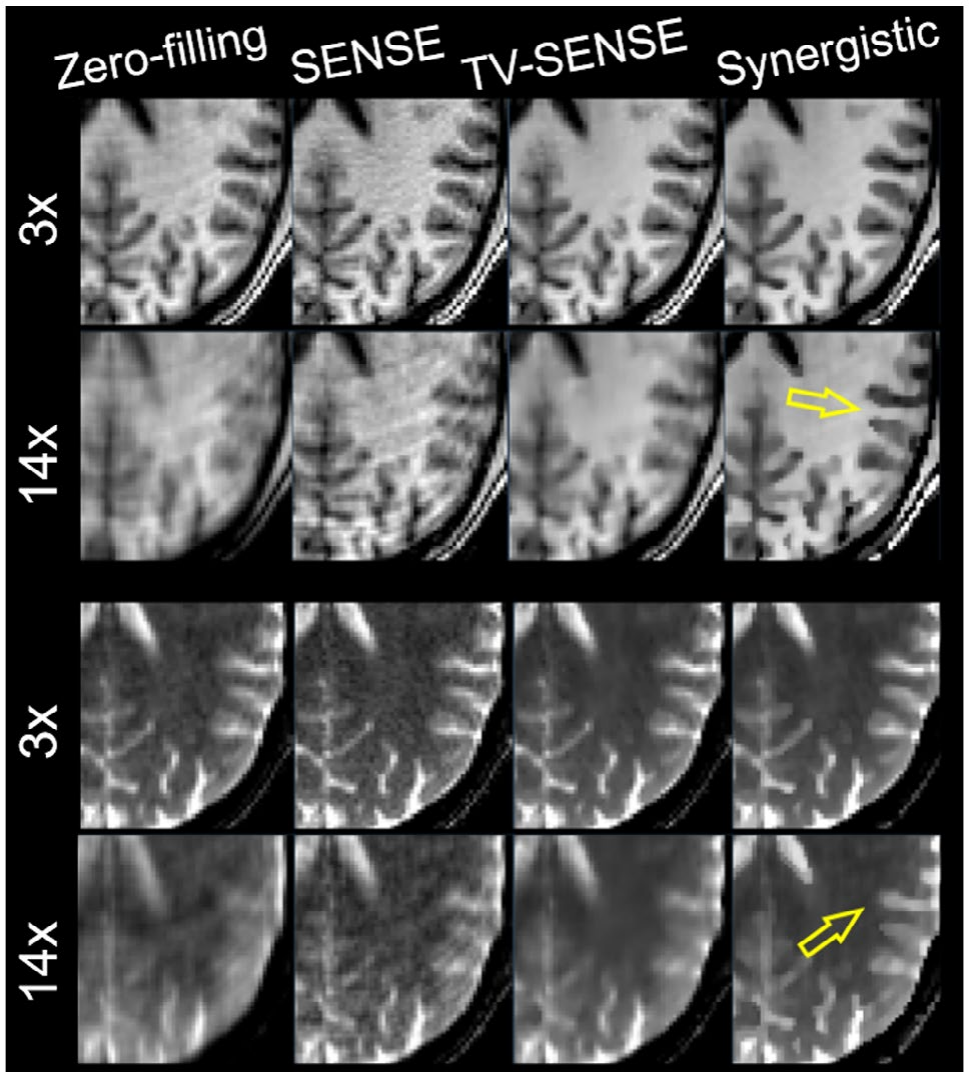
Zoomed‐in of Figure 5.

**Figure 7:**
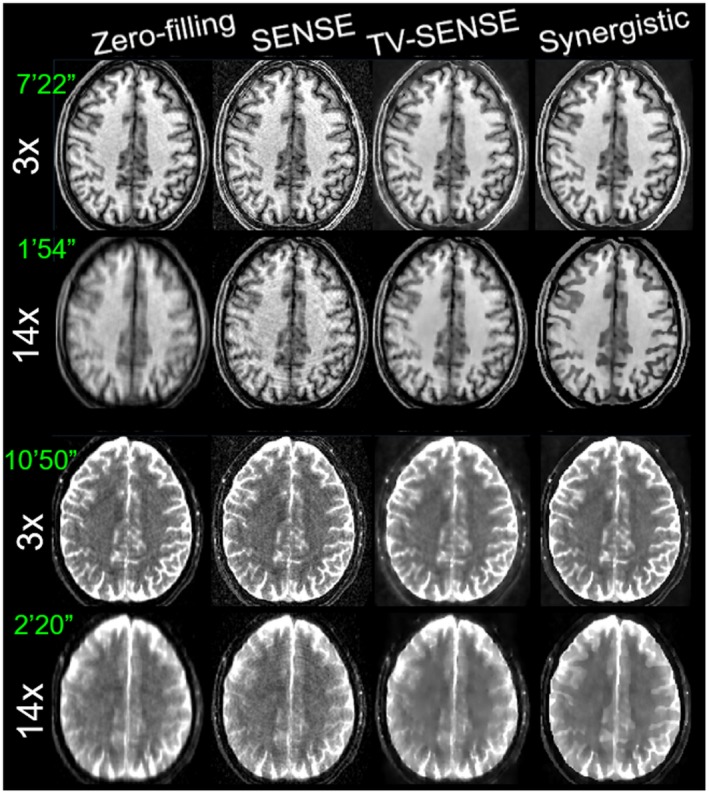
Same as Figure 5 for another healthy volunteer.

**Figure 8:**
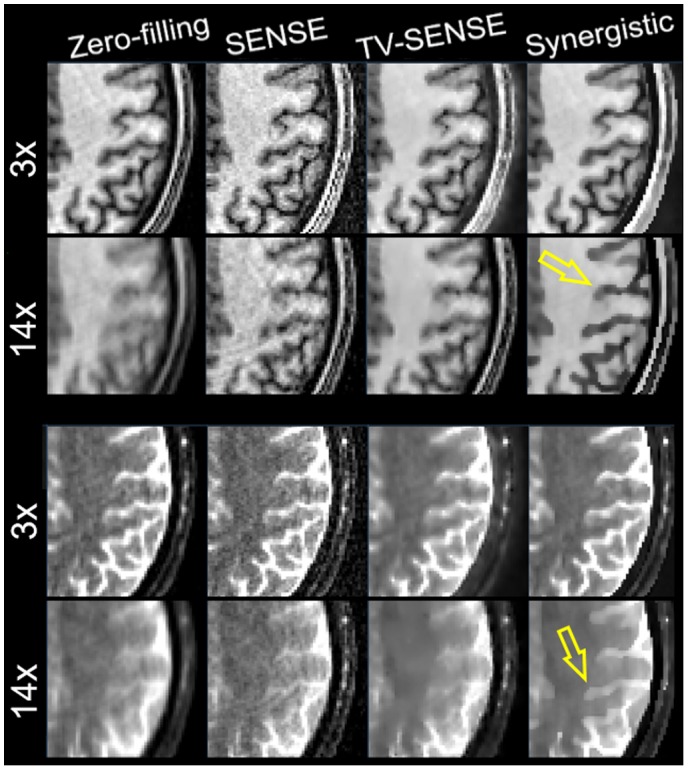
Zoomed‐in of Figure 7.

### 4.3 In vivo PET‐MR data

**Figure 9:**
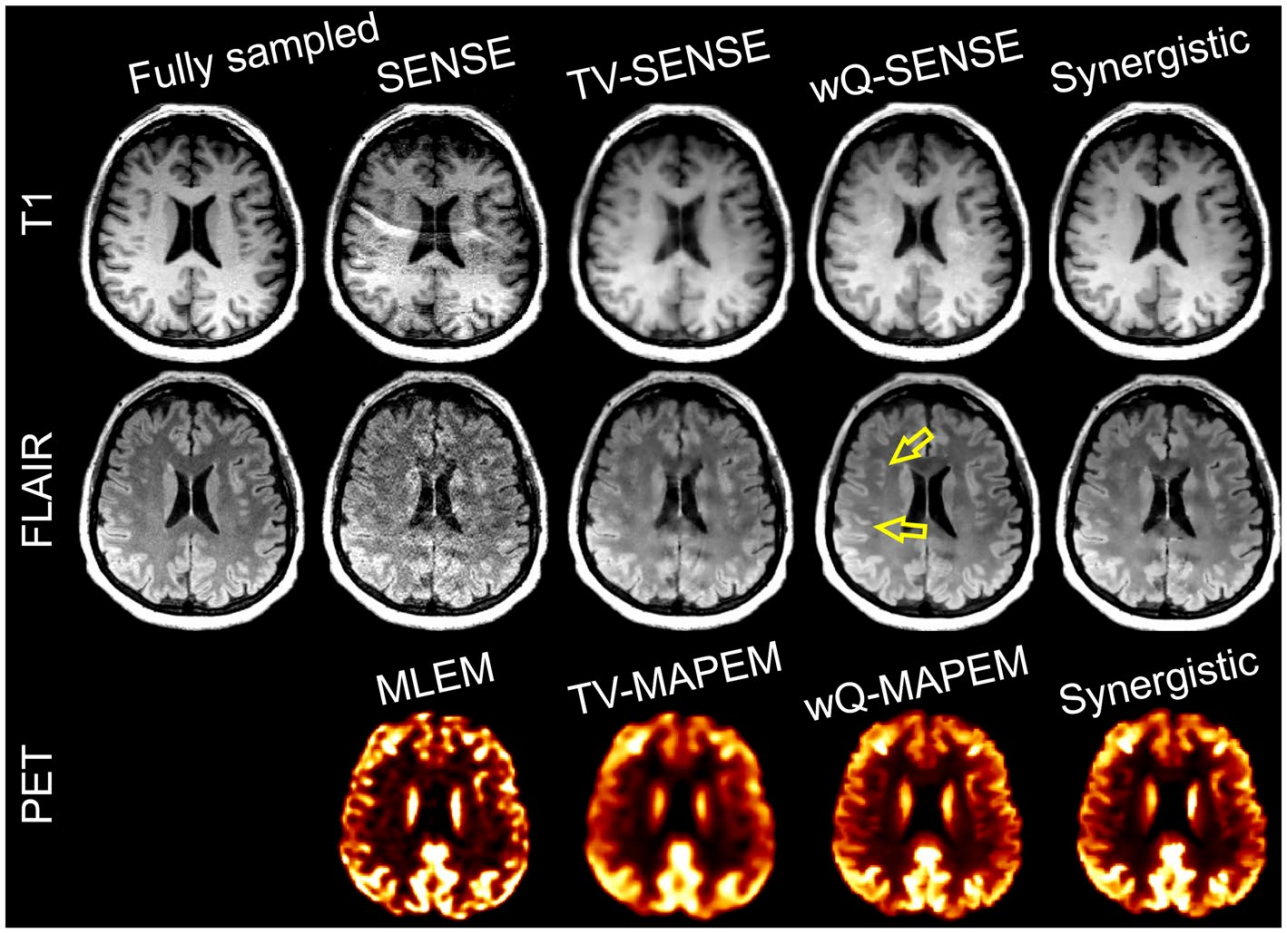
Synergistic PET‐MR image reconstruction of the PET‐MR dataset in comparison with the conventional and separate reconstruction methods.

**Figure 10:**
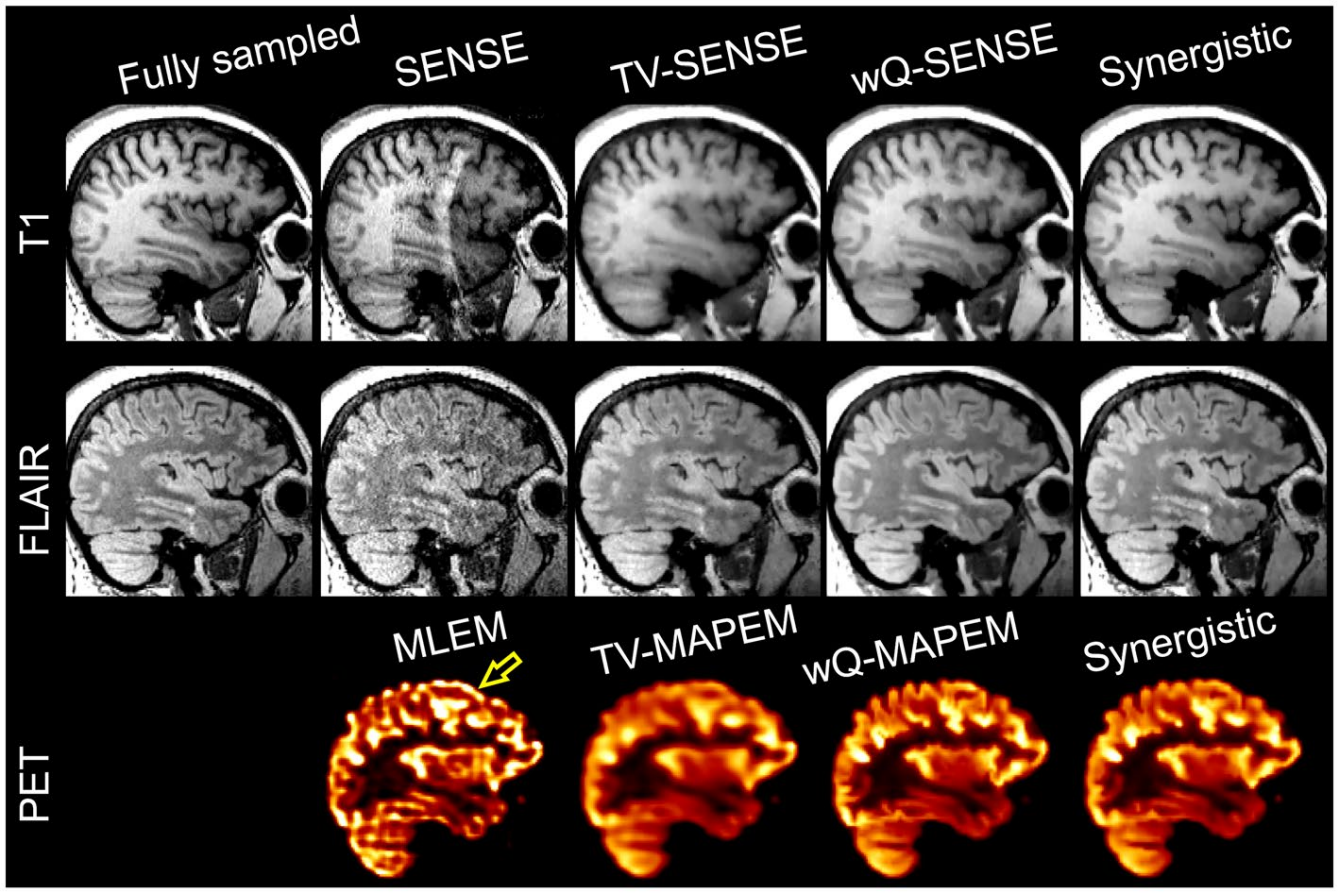
Comparison of different synergistic PET‐MR image reconstruction of the in vivo PET‐MR dataset.

Figure 9 and Supporting Information Figure S10 show the conventional and synergistic reconstruction results of the FDG‐PET/T_1_/FLAIR dataset. In this experiment, the reference MR images included the SENSE reconstruction of fully sampled T_1_ data and the 2×‐accelerated FLAIR datasets. For PET, there was no reference image. As shown, the MLEM reconstruction suffers from noise and Gibbs ringing artifacts at the edges (see arrow in Figure 10). TV reconstructions notably reduce noise and aliasing artifacts apparent in undersampled SENSE reconstructions but at the expense of resolution and detail loss. The wQ‐MAPEM and wQ‐SENSE reconstructions guided by reference T_1_ and FLAIR images improve all modalities by reducing noise and Gibbs/aliasing artifacts and can recover details. Synergistic reconstruction of PET‐MR data show that these reconstructions perform fairly comparably to the wQ‐MAPEM and wQ‐SENSE reconstructions while only using undersampled data (9× for T_1_ and 6× for FLAIR). In addition, as shown by the arrows in Figure 7 and Supporting Information Figure S11, wQ‐SENSE has introduced pseudo structures in the FLAIR image due to mismatches between the T_1_ and FLAIR images, whereas they are not present in our synergistically reconstructed FLAIR image.

Supporting Information Figure S12 compares CNR between gray and white matter for PET, T_1_, and FLAIR datasets reconstructed by different methods (not all shown in Figure 9). For PET images, the wQ‐MAPEM and SynPETMR reconstructions achieve the highest CNR, whereas the TV‐MAPEM results in the lowest CNR due to reduced contrast between the gray and white matter. For T_1_ images, the SynMR‐T_1_‐FLAIR and SynPETMR‐T_1_ methods achieve a relatively high CNR. The SynPETMR‐T_1_‐FLAIR method and wQ‐SENSE achieved similar but nonetheless lower CNR, which can be attributed to higher background noise. For the FLAIR images, the results show that almost all the reconstructions suffer from high background noise and low contrast, leading to negative CNR. However, the results show that the synergistic reconstructions exhibit a relatively better performance.

### 4.4 Benefits of PET for MR image reconstruction

Our simulation results presented in Figures 3 and 4 show that synergistic reconstruction of PET and T_1_ MR (i.e., SynPETMR‐T_1_) and that of T_1_ and T_2_ (i.e., SynMR‐T_1_‐T_2_) perform quantitatively similar. However, visual inspection of the images, as shown in Supporting Information Figure S13, reveals that the SynPETMR‐T_1_ method is outperformed by SynMR‐T_1_‐T_2_ in the recovery of structural details. That is, the T_2_ image provides more information for the T_1_ image reconstruction than for the PET reconstruction. This can be attributed to the fact that the PET data are subject to both noise and detector blurring. Figure 10 compares different synergistic reconstructions of the in vivo PET‐MR dataset. The results show that all 3 synergistic methods deliver T_1_ images of a similar quality.

### 4.5 Coupling of image modalities

The key component of synergistic reconstruction is coupling of common boundary information between different image modalities. In the proposed prior, this coupling happens through joint similarity coefficients calculated from similarity coefficients of individual images (see Equation [Link]). To demonstrate this coupling effect, we compared synergistic T_1_ and T_2_ MR reconstructions with those guided using individual similarity coefficients, namely *self‐guided SENSE*. It is worth mentioning that the difference between self‐guided SENSE and wQ‐SENSE is that in the former the weights are iteratively calculated from each image itself, whereas in the latter they are precomputed from a prior high‐quality image. Supporting Information Figure S14 shows the results of the brain phantom for different reconstruction methods. As indicated by the yellow arrow, the wQ‐SENSE reconstruction of the T_1_ image, guided by a fixed fully sampled T_2_ image, suppresses the lesion, whereas the self‐guided SENSE and synergistic methods preserve this unique lesion. However, the white arrows indicate that the self‐guided method cannot recover the pointed structures, whereas the synergistic method is able to fairly recover them due to coupling of T_1_ and T_2_ boundary information. Supporting Information Figure S15 also highlights this for synergistic reconstruction of a 14×‐accelerated in vivo MR dataset. As indicated by the arrow, for reconstruction of the T_2_ image the synergistic reconstruction outperforms the self‐guided SENSE reconstruction.

### 4.6 Comparison with previous methods

In Ref. 3, we had previously proposed a generalized joint TV prior with an iterative rescaling of PET and MR gradients using a single global factor. Despite the promising simulation results, this rescaling method is not a robust solution in a practical setting because the edges have different magnitudes for in vivo PET‐MR images. Thus, a single scale factor cannot scale them properly, and the joint prior may degenerate to a separate conventional prior. In Supporting Information Figure S16, we compare our previous and current methods using a resolution phantom devised by Ref. 1 as a benchmark. As shown, both methods improve the quality of the PET‐MR images, especially the joint TV prior, despite the large relative intensity differences within the PET‐MR data. However, comparing our in vivo reconstructions in Ref. 3 with those in the present work demonstrates that our newly proposed method can effectively harness the synergy of PET‐MR data.

We also compared our method with the joint TGV method using the phantom and software publicly provided by Ref. 2. The results presented in Supporting Information Figure S17 show that both synergistic methods perform similarly for modality‐shared edges but differently for modality‐unique lesions. For the PET‐unique lesion, the TGV algorithm resulted in enhancement of the lesion, whereas our proposed method performs similarly to the standard MLEM. The TGV enhancement can be attributed to the fact that TGV relies on a total variation prior, which is an edge‐preserving prior. For this reason, the TGV PET reconstruction appears also nonuniform and patchy. For an MR‐unique lesion, the TGV results show that the MR unique lesion has been transferred into the PET image, which is not the case for our method. Our proposed reweighted quadratic prior can be easily extended to a that of a TV; however, it will make the optimization more complicated because the TV prior is not continuously differentiable. Thus, either an advanced optimization algorithm is required or a smoothed TV prior (with an additional hyperparameter that controls the degree of edge preservation).

## 5 DISCUSSION

This work presents a reconstruction methodology that addresses some of the major challenges of synergistic reconstruction while still exploiting the synergy of PET‐MR data. The proposed prior has the following properties: First, it is independent of the intensity ranges of the individual images (i.e., in Figure 1, the T_1_ image is in the range [0, 3 × 10^4^], and the PET image is in [0, 1]). This is because the kernels (G) defined in Equation [Link] are always in [0, 1], according to the definition of a normal distribution. Therefore, the joint coefficients (ω) are not dominated by the image with larger signal magnitude (in the above example, the T_1_ image) because all kernels are normalized and thus will have equal contribution to the joint coefficients. Second, it is scale‐invariant, that is, if the intensity range of a given image varies by a factor (i.e., if the T_1_ image in Figure 1 is scaled from [0, 3 × 10^4^] to [0, 3]), the kernels remain identical. This is because the images are first normalized to [0, 1], and then the kernels are calculated. Third, it is contrast‐invariant; that is, if the intensity range of a given image is inverted, the kernels remain identical due to the square in the exponent of the normal distribution. Note that the third property is not due to the nature of the proposed method but the preprocessing step of normalizing the intensity ranges of all input signals. In addition, the third property also holds true for any gradient‐based coupling such as joint TV or joint TGV reconstruction.

Given the high computational expense of synergistic reconstruction, we heuristically selected the required hyperparameters, as summarized in Supporting Information Table S3. For the simulations, 1 global iteration of synergistic PET‐T_1_‐T_2_ reconstruction, which consisted of 2 MAPEM iterations for PET, 2 CG iterations for T_1_, and 2 CG iterations for T_2_ (all with neighborhood sizes of 5 × 5 × 5) and their corresponding resampling, took about 8 min. Hence, 500 global iterations led to a total reconstruction time of 2.7 days. For in vivo PET‐MR data using the same update schedule as the simulations but with a smaller neighborhood size of 3 × 3 × 3 (to reduce computation time), 1 global iteration took about 4.3 min (total 11.25 h for 150 global iterations).

Overall, the performance of synergistic reconstruction depends on the selected hyperparameters, especially the β and σ used in Equations (2) and [Link]. The hyperparameter β controls the level of smoothness, whereas σ controls the level of edge preservation. For a given β, as σ is decreased all the details of the reconstructed images, including noise and undersampling artifacts, will be preserved because the resulting Gaussian similarity function will be narrower and will map a smaller weight to intensity differences between voxels. Smaller weights lead to reduced regularization. In our experience, β has a higher impact on the algorithms' performance and varies substantially for different datasets. Neighborhood size is also another hyperparameter. It has been recommended that larger neighborhoods lead to better performance of guided reconstruction^33^; however, in this work we noticed that a neighborhood size of 3 × 3 × 3 is sufficient for both edge identification and faster computation. The update schedule is also another influential hyperparameter. The convergence rate and path of PET and MR reconstructions are different because the PET system matrix and MR encoding matrix have different condition numbers and use different optimization algorithms. The key component of our methodology is calculation of the mutual similarity coefficients from the current PET and MR image estimates; hence, the convergence of PET‐MR reconstructions per global iteration is of importance. In particular, if the MR undersampling factor is low, the convergence of the CG algorithm is faster. In this work, the updates for PET and MR reconstructions were kept at 2 to 4 iterations per global iterations. Moreover, convergence of the employed alternating optimization to a global maximum is unknown because this algorithm was mainly built on separate PET and MR reconstruction methods. Hence, further work is required to study convergence.

In this work, we used a reweighted quadratic prior instead of a TV or TGV prior. The prior does not introduce additional hyperparameters or constant factors, and thanks to its continuous differentiability there is no need for advanced optimization algorithms, which in turn would add extra hyperparameters to ensure convergence. However, the iterative calculation of weighting coefficients means that the proposed joint objective has multiple local maxima because different initializations will result in different weighting coefficients and thus different solutions. Hence, our objective function is nonconvex (strictly speaking, nonconcave).

In terms of computational complexity compared to previous methods, the added computational load of our algorithm is not substantial because there is no need for optimization with respect to primal and dual variables as used in Ref. 2; and there is no optimization of an augmented Lagrangian problem as used in Ref. 3. The most time‐consuming stages are the individual regularized PET and MR reconstructions. However, the notable added computational burden of our algorithm is the spatial transformations used for mapping different image contrasts to each other before joint calculation of the weighting coefficients. We opted for this extra computational cost to reconstruct images in their native resolution. Knoll el al.^2^ performed reconstruction of PET‐MR images in the space of the MR image with high resolution, which can be especially time‐consuming for PET reconstruction.

Our in vivo data results in Figure 9 demonstrated that synergistic reconstruction can improve the quality of PET‐MR images even compared to guided reconstructions, which utilize fully sampled MR images. For synergistic reconstructions, it was found that the gain obtained by PET is more than the little, if any, gain obtained for the MR images from the PET data. This can be mainly attributed to the lower resolution and relatively high noise level of PET data.

To be readily comparable to in vivo datasets, in this study the simulated fully sampled MR reconstructions were considered as the reference images instead of the ground truth. This is because it is widely regarded within the MR community that the goal is to reconstruct an MR image from undersampled data with a quality comparable to a fully sampled MR image. In contrast, for PET imaging there is no such reference image due to the limited acquisition time and the limited resolution of current clinical PET scanners.

In this study, the feasibility and benefits of synergistic multi‐contrast MR and PET images was demonstrated. Future work will require evaluation of synergistic reconstruction of non‐FDG PET and MR data. In a normal healthy brain, FDG often has a uniform but contrasting uptake in gray and white matter, following the anatomical patterns of the MR images. For non‐FDG tracers, the potential merit of synergistic PET‐MR reconstruction would still need to be demonstrated because such tracers might demonstrate a local uptake without any specific anatomical correspondence. Future work should also include synergistic reconstruction of multi‐frame dynamic PET data for improved SNR of the corresponding image frames.

## 6 CONCLUSION

The proposed method aims to exploit the synergy of PET‐MR or multi‐contrast MR images irrespective of their relative intensity differences and contrasts. Mutually weighted quadratic priors were exploited to promote the simplicity and stability of the resulting algorithm. Our simulations and in vivo data reconstructions showed that the proposed synergistic reconstruction can considerably improve on existing TV regularization methods and even prior‐image guided reconstructions, particularly in the presence of mismatches between image modalities.

## Supporting information

TABLE S1 Abbreviations and descriptions of the reconstruction algorithms used for the simulated and real datasets in this study.
TABLE S2. Reconstruction set‐ups for k‐space undersampling of different MR image contrasts of the simulation and clinical datasets
TABLE S3. Parameters used for the reconstruction of the simulated and in‐vivo datasets using the studied reconstruction methods.
FIGURE S1. Background ROIs used for calculation of CNR in the simulated brain phantom.
FIGURE S2. Same as Figure 1, but with added voxel‐wise error maps.
FIGURE S3. Zoomed‐in of Figure 1.
FIGURE S4. Same as Figure 2, but with added voxel‐wise error maps.
FIGURE S5. Zoomed‐in of Figure 2.
FIGURE S6. Convergence of the reconstruction methods in terms of normalized root mean square error (NRMSE) in the whole brain for each image update of the simulated PET, T1 and T2 MR datasets.
FIGURE S7. Synergistic reconstruction of the prospectively undersampled T1 (left) and T2 (right) datasets for the first healthy volunteer. The acceleration factor and resulting acquisition time (in minutes and seconds) of each scan are shown.
FIGURE S8. Synergistic reconstruction of the prospectively undersampled T1 (left) and T2 (right) datasets for the second healthy volunteer. Acceleration factor and resulting acquisition time (in minutes and seconds) of each scan are shown.
FIGURE S9. CNR between grey and white matter of the T1 and T2 images of the in‐vivo MR datasets.
FIGURE S10. Same as Figure 9 but for a sagittal slice. The arrow indicates Gibbs artefacts in the PET MLEM reconstruction.
Figure S11. Zoomed in from Figure 9. The arrows point to structural artefacts induced by T1‐guidance of the FLAIR reconstruction, (i.e. wQ‐SENSE).
FIGURE S12. CNR between grey and white matter of the FDG‐PET, T1 and FLAIR images of the in‐vivo PET‐MR dataset.
FIGURE S13. Comparison of different synergistic reconstructions of the simulated PET‐MR dataset for synergistic reconstruction of T1 and T2 (SynMR‐T1‐T2), PET and T1 (SynPETMR‐T1), and PET, T1 and T2 (SynPETMR‐T1‐T2). Comparison of SynMR‐T1‐T2 and SynPET‐MR‐T1 shows that the T1 image has been improved more when synergistically reconstructed with the T2 image than the PET image (see arrows). Synergistic reconstruction of all data together (in SynPETMR‐T1‐T2) is beneficial for all reconstructions.
FIGURE S14. Illustration of the coupling effect of common boundary information between T1 and T2 datasets through their synergistic reconstruction. Compared to self‐guided reconstruction, the synergistic one is able to recover more structural details and at the same time preserve unique lesions that are otherwise suppressed by wQ‐SENSE.
FIGURE S15. Same as Supporting Figure 14, but for the volunteer MR scan #2.
FIGURE S16. Performance comparison of the proposed synergistic reconstruction method with our previous work in (3), using a joint total variation prior generalized using a non‐convex potential function, on the resolution phantom proposed by Ehrhardt et al (1) for the ‘*radial 20*' simulation set‐up.
FIGURE S17. Performance comparison of our proposed synergistic algorithm with the synergistic TGV one proposed in Ref (2). In this comparison, the code and simulated dataset were obtained from Ref. (24).

## References

[1] Ehrhardt MJ , Thielemans K , Pizarro L , et al. Joint reconstruction of PET‐MRI by exploiting structural similarity. Inverse Probl. 2014;31:15001.

[2] Knoll F , Holler M , Koesters T , Otazo R , Bredies K , Sodickson DK . Joint MR‐PET reconstruction using a multi‐channel image regularizer. IEEE Trans Med Imaging. 2017;36:1–16. 28055827 10.1109/TMI.2016.2564989 PMC5218518

[3] Mehranian A , Belzunce M , Prieto C , Hammers A , Reader A . Synergistic PET and SENSE MR image reconstruction using joint sparsity regularization. IEEE Trans Med Imaging. 2018;37:20–34. 28436851 10.1109/TMI.2017.2691044

[4] Bowsher JE , Hong Y , Hedlund LW , et al. Utilizing MRI information to estimate F18‐FDG distributions in rat flank tumors. In: IEEE Symposium Conference Record Nuclear Science. Rome, Italy, 2004; Vol. 4: 2488–2492.

[5] Kazantsev D , Lionheart WRB , Withers PJ , Lee PD . Multimodal image reconstruction using supplementary structural information in total variation regularization. Sens Imaging. 2014;15:97. 25484635 10.1007/s11220-014-0097-5 PMC4247493

[6] Mehranian A , Belzunce MA , Niccolini F , et al. PET image reconstruction using multi‐parametric anato‐functional priors. Phys Med Biol. 2017;62:5975–6007. 28570263 10.1088/1361-6560/aa7670

[7] McGibney G , Smith MR , Nichols ST , Crawley A . Quantitative evaluation of several partial Fourier reconstruction algorithms used in MRI. Magn Reson Med. 1993;30:51–59. 8371675 10.1002/mrm.1910300109

[8] Pruessmann KP , Weiger M , Scheidegger MB , Boesiger P . SENSE: sensitivity encoding for fast MRI. Magn Reson Med. 1999;42:952–962. 10542355

[9] Ying L , Liu B , Steckner MC , Wu G , Wu M , Li S‐J . A statistical approach to SENSE regularization with arbitrary k ‐space trajectories. Magn Reson Med. 2008;60:414–421. 18666100 10.1002/mrm.21665

[10] Liu B , King K , Steckner M , Xie J , Sheng J , Ying L . Regularized sensitivity encoding (SENSE) reconstruction using Bregman iterations. Magn Reson Med. 2009;61:145–152. 19097223 10.1002/mrm.21799

[11] Candès EJ , Wakin MB , Boyd SP . Enhancing sparsity by reweighted ℓ1 minimization. J Fourier Anal Appl. 2008;14:877–905.

[12] Otazo R , Candès E , Sodickson DK . Low‐rank plus sparse matrix decomposition for accelerated dynamic MRI with separation of background and dynamic components. Magn Reson Med. 2015;73:1125–1136. 24760724 10.1002/mrm.25240 PMC4207853

[13] Kreutz‐Delgado K , Murray JF , Rao BD , Engan K , Lee T‐W , Sejnowski TJ . Dictionary learning algorithms for sparse representation. Neural Comput. 2003;15:349–396. 12590811 10.1162/089976603762552951 PMC2944020

[14] Ehrhardt MJ , Betcke MM . Multi‐contrast MRI reconstruction with structure‐guided total variation. SIAM J Imaging Sci. 2016;9:1084–1106.

[15] Birns S , Kim B , Ku S , Stangl K , Needell D . A practical study of longitudinal reference based compressed sensing for MRI. 2016 arXiv:1608.04728.

[16] Weizman L , Eldar YC , Bashat DB . Compressed sensing for longitudinal MRI: an adaptive‐weighted approach. Med Phys. 2015;42:5195. 26328970 10.1118/1.4928148

[17] Weizman L , Eldar YC , Eilam A , Londner S , Artzi M , Ben BD . Fast reference‐based MRI. In: 2015 37th Annual International Conference of the IEEE Engineering in Medicine and Biology Society (EMBC), Milan, Italy, 2015 p. 7486–7489. 10.1109/EMBC.2015.7320123 26738023

[18] Peng X , Du H‐Q , Lam F , Babacan SD , Liang Z‐P . Reference‐driven MR image reconstruction with sparsity and support constraints. In: 2011 IEEE International Symposium on Biomedical Imaging: From Nano to Macro. ISBI'11. 2011 p. 89–92.

[19] Chavarrías C , Abascal JFPJ , Montesinos P , Desco M . Exploitation of temporal redundancy in compressed sensing reconstruction of fMRI studies with a prior‐based algorithm (PICCS). Med Phys. 2015;42:3814–3821. 26133583 10.1118/1.4921365

[20] Huang J , Chen C , Axel L . Fast multi‐contrast MRI reconstruction. Magn Reson Imaging. 2014;32:1344–1352. 25193110 10.1016/j.mri.2014.08.025

[21] Weizman L , Mota JFC , Song P , Eldar YC , Rodrigues MRD . Joint multicontrast MRI reconstruction. In: 6th Signal Processing with Adaptive Sparse Structured Representations Workshop, Lisbon, Portugal, 2017.

[22] Chen C , Li Y , Huang J . Calibrationless parallel MRI with joint total variation regularization. Med Image Comput Comput Assist Interv. 2013;106–114. 10.1007/978-3-642-40760-4_14 24505750

[23] Bilgic B , Goyal VK , Adalsteinsson E . Multi‐contrast reconstruction with Bayesian compressed sensing. Magn Reson Med. 2011;66:1601–1615. 21671267 10.1002/mrm.22956 PMC3175273

[24] Holler M , Huber RM , Knoll F . Coupled regularization with multiple data discrepancies. Inverse Probl. 2018. doi: 10.1088/1361-6420/aac539. PMC6344056 30686851

[25] Ramani S , Fessler JA . Parallel MR image reconstruction using augmented Lagrangian methods. IEEE Trans Med Imaging. 2011;30:694–706. 21095861 10.1109/TMI.2010.2093536 PMC3081617

[26] De Pierro AR . A modified expectation maximization algorithm for penalized likelihood estimation in emission tomography. IEEE Trans Med Imaging. 1995;14:132–137. 18215817 10.1109/42.370409

[27] Wang G , Qi J . Penalized likelihood PET image reconstruction using patch‐based edge‐preserving regularization. IEEE Trans Med Imaging. 2012;31:2194–2204. 22875244 10.1109/TMI.2012.2211378 PMC4080915

[28] Pruessmann KP , Weiger M , Börnert P , Boesiger P . Advances in sensitivity encoding with arbitrary k‐space trajectories. Magn Reson Med. 2001;46:638–651. 11590639 10.1002/mrm.1241

[29] Collins DL , Zijdenbos AP , Kollokian V , et al. Design and construction of a realistic digital brain phantom. IEEE Trans Med Imaging. 1998;17:463–468. 9735909 10.1109/42.712135

[30] Belzunce MA , Mehranian A , Chalampalakis Z , Reader AJ . Evaluation of shift‐invariant image‐space PSFs for the Biograph mMR PET scanner. In PSMR Conference. 2017.

[31] Guerquin‐Kern M , Lejeune L , Pruessmann KP , Unser M . Realistic analytical phantoms for parallel magnetic resonance imaging. IEEE Trans Med Imaging. 2012;31:626–636. 22049364 10.1109/TMI.2011.2174158

[32] Bustin A , Ginami G , Cruz G , et al. Five‐minute whole‐heart coronary MRA with sub‐millimeter isotropic resolution, 100% respiratory scan efficiency and 3D‐PROST reconstruction. Magn Reson Med. 2018. doi: 10.1002/mrm.27354. PMC6617822 30058252

[33] Vunckx K , Atre A , Baete K , et al. Evaluation of three MRI‐based anatomical priors for quantitative PET brain imaging. IEEE Trans Med Imaging. 2012;31:599–612. 22049363 10.1109/TMI.2011.2173766

