# Supplementary material for: Multi‐modal synergistic PET and MR reconstruction using mutually weighted quadratic priors
Source: Magn Reson Med. 2018 Oct 16;81(3):2120–34. doi: 10.1002/mrm.27521 (PMC6563465; doi:10.1002/mrm.27521)
Supplement: Supplementary file 1 — TABLE S1 Abbreviations and descriptions of the reconstruction algorithms used for the simulated and real datasets in this study. TABLE S2. Reconstruction set‐ups for k‐space undersampling of different MR image contrasts of the simulation and clinical datasets TABLE S3. Parameters used for the reconstruction of the simulated and in‐vivo datasets using the studied reconstruction methods. FIGURE S1. Background ROIs used for calculation of CNR in the simulated brain phantom. FIGURE S2. Same as Figure 1, but with added voxel‐wise error maps. FIGURE S3. Zoomed‐in of Figure 1. FIGURE S4. Same as Figure 2, but with added voxel‐wise error maps. FIGURE S5. Zoomed‐in of Figure 2. FIGURE S6. Convergence of the reconstruction methods in terms of normalized root mean square error (NRMSE) in the whole brain for each image update of the simulated PET, T1 and T2 MR datasets. FIGURE S7. Synergistic reconstruction of the prospectively undersampled T1 (left) and T2 (right) datasets for the first healthy volunteer. The acceleration factor and resulting acquisition time (in minutes and seconds) of each scan are shown. FIGURE S8. Synergistic reconstruction of the prospectively undersampled T1 (left) and T2 (right) datasets for the second healthy volunteer. Acceleration factor and resulting acquisition time (in minutes and seconds) of each scan are shown. FIGURE S9. CNR between grey and white matter of the T1 and T2 images of the in‐vivo MR datasets. FIGURE S10. Same as Figure 9 but for a sagittal slice. The arrow indicates Gibbs artefacts in the PET MLEM reconstruction. Figure S11. Zoomed in from Figure 9. The arrows point to structural artefacts induced by T1‐guidance of the FLAIR reconstruction, (i.e. wQ‐SENSE). FIGURE S12. CNR between grey and white matter of the FDG‐PET, T1 and FLAIR images of the in‐vivo PET‐MR dataset. FIGURE S13. Comparison of different synergistic reconstructions of the simulated PET‐MR dataset for synergistic reconstruction of T1 and T2 (SynMR‐T1‐T2), PET and T1 [file MRM-81-2120-s001.docx]

**Supporting Materials**

**Supporting Information Table S1**. Abbreviations and descriptions of the reconstruction algorithms used for the simulated and real datasets in this study.

| Modality | Algorithm | Description |
| --- | --- | --- |
| PET | MLEM | Eq.[5] |
|  | TV-MAPEM | MAPEM recon. using TV prior and the one-step-late algorithm (33). |
|  | wQ-MAPEM | MAPEM recon. using quadratic prior weighted by a fully sampled MR image, Eq.[5-6]. |
|  |  |  |
| MR | SENSE | SENSE recon. of fully or undersampled MR data using the CG algorithm (28). |
|  | TV-SENSE | SENSE recon. of undersampled data using TV prior and alternating direction method of multipliers algorithm (3). |
|  | wQ-SENSE | SENSE recon. of undersampled data using a quadratic prior weighted by a fully sampled MR image and the CG algorithm. |
|  | SynMR-T1-T2(FLAIR^†^) | Synergistic recon. of undersampled T1 and undersampled T2 (or FLAIR) images using steps 2 and 3b of proposed algorithm (Table 1) |
|  |  |  |
| PET-MR | SynPETMR-T1(T2) | Synergistic recon. of PET and undersampled T1(or T2) images |
|  | SynPETMR-T1-T2 (FLAIR) | Synergistic recon. of PET, undersampled T1 and undersampled T2 (or FLAIR) images |

† FLAIR : Fluid attenuated inversion recovery

**Supporting Information Table S2**. Reconstruction set-ups for k-space undersampling of different MR image contrasts of the simulation and clinical datasets

| **Simulated PET-MR data** |  | **In-vivo MR dataset for** |  | **In-vivo PET-MR dataset** |
| --- | --- | --- | --- | --- |
| T1, Cartesian under-sampling in phase encoding direction, R = 6 |  | Both T1 and T2 datasets VD-CASPR with under-sampling factors of 3, 9 and 14 |  | T1, Cartesian under-sampling in phase (R = 3) and slice encoding (R = 3) directions, total R = 9 |
| T2, Radial under-sampling with stack of 20 spokes (R ~10) |  |  |  | FLAIR, Cartesian under-sampling in phase (R = 2) and slice encoding (R = 3) directions, total R = 6 |

**Supporting Information Table S3**. Parameters used for the reconstruction of the simulated and in-vivo datasets using the studied reconstruction methods.

|  |  |  | PET | | |  | T1 | | |  | T2/FLAIR | | |
| --- | --- | --- | --- | --- | --- | --- | --- | --- | --- | --- | --- | --- | --- |
|  | Reconstruction method | No. global iterations | No. MAPEM iterations ($P_{iter}$) | $\beta$ | $\sigma$ |  | No. SENSE-CG iterations ($M_{iter}$) | $\beta$ | $\sigma$ |  | No. SENSE-CG iterations | $\beta$ | $\sigma$ |
| Simulation | *PET*: MLEM | – | 1000 | 0 | – |  | – | – | – |  | – | – | – |
|  | *PET*: TV-MAPEM | – | 1000 | 100 | – |  | – | – | – |  | – | – | – |
|  | *PET*: wQ-MAPEM | – | 1000 | 6×10^5^ | 0.08 |  | – | – | – |  | – | – | – |
|  |  |  |  |  |  |  |  |  |  |  |  |  |  |
|  | *T1*: Fully sampled | – | – | – | – |  | 150 | – | – |  | – | – | – |
|  | *T1*: SENSE | – | – | – | – |  | 150 | – | – |  | – | – | – |
|  | *T1*: TV-SENSE | 600 | – | – | – |  | 2 | 15 | – |  | – | – | – |
|  | *T1*: wQ- SENSE | – | – | – | – |  | 1000 | 0.05 | 0.01 |  | – | – | – |
|  |  |  |  |  |  |  |  |  |  |  |  |  |  |
|  | *T2*: Fully sampled | – | – | – | – |  | – | – | – |  | 150 | – | – |
|  | *T2*: SENSE | – | – | – | – |  | – | – | – |  | 150 | – | – |
|  | *T2*: TV-SENSE | 600 | – | – | – |  | – | – | – |  | 2 | 15 | – |
|  | *T2*: wQ-SENSE | – | – | – | – |  | – | – | – |  | 1000 | 2 | 0.05 |
|  |  |  |  |  |  |  |  |  |  |  |  |  |  |
|  | SynMR-T1-T2 | 500 | – | – | – |  | 2 | 0.3 | 0.03 |  | 2 | 0.3 | 0.03 |
|  | SynPETMR-T1 | 500 | 2 | 6×10^5^ | 0.1 |  | – | – | – |  | 2 | 0.4 | 0.02 |
|  | SynPETMR-T1-T2 | 500 | 2 | 6×10^5^ | 0.1 |  | 2 | 0.4 | 0.02 |  | 2 | 0.4 | 0.02 |
|  |  |  |  |  |  |  |  |  |  |  |  |  |  |
| Real MR data : Volunteer #1 | *T1:* SENSE (3x) | – | – | – | – |  | 5 | – | – |  | – | – | – |
|  | *T1:* SENSE (9x) | – | – | – | – |  | 5 | – | – |  | – | – | – |
|  | *T1:* SENSE (14x) | – | – | – | – |  | 5 | – | – |  | – | – | – |
|  | *T1:* TV-SENSE (3x) | 20 | – | – | – |  | 2 | 0.001 | – |  | – | – | – |
|  | *T1:* TV-SENSE (9x) | 20 | – | – | – |  | 2 | 0.001 | – |  | – | – | – |
|  | *T1:* TV-SENSE (14x) | 20 | – | – | – |  | 2 | 0.001 | – |  | – | – | – |
|  |  |  |  |  |  |  |  |  |  |  |  |  |  |
|  | *T2:* SENSE (3x) | – | – | – | – |  | 5 | – | – |  | – | – | – |
|  | *T2:* SENSE (9x) | – | – | – | – |  | 5 | – | – |  | – | – | – |
|  | *T2:* SENSE (14x) | – | – | – | – |  | 5 | – | – |  | – | – | – |
|  | *T2:* TV-SENSE (3x) | 20 | – | – | – |  | 2 | 0.001 | – |  | – | – | – |
|  | *T2:* TV-SENSE (9x) | 20 | – | – | – |  | 2 | 0.001 | – |  | – | – | – |
|  | *T2:* TV-SENSE (14x) | 20 | – | – | – |  | 2 | 0.001 | – |  | – | – | – |
|  |  |  |  |  |  |  |  |  |  |  |  |  |  |
|  | SynMR-T1-T2 (3x) | 20 | – | – | – |  | 4 | 20 | 0.03 |  | 4 | 20 | 0.03 |
|  | SynMR-T1-T2 (9x) | 20 | – | – | – |  | 8 | 15 | 0.03 |  | 8 | 15 | 0.03 |
|  | SynMR-T1-T2 (14x) | 20 | – | – | – |  | 10 | 7 | 0.03 |  | 10 | 7 | 0.03 |
|  |  |  |  |  |  |  |  |  |  |  |  |  |  |
| Real MR data: Volunteer #2 | *T1:* SENSE (3x) |  | – | – | – |  | 5 | – | – |  | – | – | – |
|  | *T1:* SENSE (9x) |  | – | – | – |  | 5 | – | – |  | – | – | – |
|  | *T1:* SENSE (14x) |  | – | – | – |  | 5 | – | – |  | – | – | – |
|  | *T1:* TV-SENSE (3x) | 10 | – | – | – |  | 2 | 0.1 | – |  | – | – | – |
|  | *T1:* TV-SENSE (9x) | 10 | – | – | – |  | 2 | 0.1 | – |  | – | – | – |
|  | *T1:* TV-SENSE (14x) | 10 | – | – | – |  | 2 | 0.05 | – |  | – | – | – |
|  |  |  |  |  |  |  |  |  | – |  |  | – | – |
|  | *T2:* SENSE (3x) |  | – | – | – |  | – | – | – |  | 5 | – | – |
|  | *T2:* SENSE (9x) |  | – | – | – |  | – | – | – |  | 5 | – | – |
|  | *T2:* SENSE (14x) |  | – | – | – |  | – | – | – |  | 5 | – | – |
|  | *T2:* TV-SENSE (3x) | 10 | – | – | – |  | – | – | – |  | 2 | 0.2 | – |
|  | *T2:* TV-SENSE (9x) | 10 | – | – | – |  | – | – | – |  | 2 | 0.2 | – |
|  | *T2:* TV-SENSE (14x) | 10 | – | – | – |  | – | – | – |  | 2 | 0.05 | – |
|  |  |  |  |  |  |  |  |  |  |  |  |  |  |
|  | SynMR-T1-T2 (3x) | 20 | – | – | – |  | 4 | 15 | 0.03 |  | 4 | 15 | 0.03 |
|  | SynMR-T1-T2 (9x) | 20 | – | – | – |  | 4 | 15 | 0.03 |  | 4 | 15 | 0.03 |
|  | SynMR-T1-T2 (14x) | 20 | – | – | – |  | 4 | 30 | 0.03 |  | 4 | 30 | 0.03 |
|  |  |  |  |  |  |  |  |  |  |  |  |  |  |
| Real PET-MR data | *PET*: MLEM | – | 300 | – | – |  | – | – | – |  | – | – | – |
|  | *PET*: TV-MAPEM | – | 300 | 100 | – |  | – | – | – |  | – | – | – |
|  | *PET*: wQ-MAPEM | – | 300 | 5×10^4^ | 0.1 |  | – | – | – |  | – | – | – |
|  |  |  |  |  |  |  |  |  |  |  |  |  |  |
|  | *T1:* Fully sampled | – | – | – | – |  | 150 | – | – |  | – | – | – |
|  | *T1:* SENSE | – | – | – | – |  | 150 | – | – |  | – | – | – |
|  | *T1:* TV-SENSE | 150 | – | – | – |  | 2 | 300 | – |  | – | – | – |
|  |  |  |  |  |  |  |  |  |  |  |  |  |  |
|  | *FLAIR:* SENSE (2x) | – | – | – | – |  | – | – | – |  | 150 | – | – |
|  | *FLAIR:* SENSE (6x) | – | – | – | – |  | – | – | – |  | 150 | – | – |
|  | *FLAIR:* TV-SENSE | 150 | – | – | – |  | – | – | – |  | 2 | 300 | – |
|  |  |  |  |  |  |  |  |  |  |  |  |  |  |
|  | SynMR-T1-FLAIR | 150 | – | – | – |  | 2 | 10 | 0.04 |  | 2 | 8 | 0.005 |
|  | SynPETMR-T1 | 150 | 2 | 3×10^4^ | 0.1 |  | 2 | 3 | 0.015 |  | – | – | – |
|  | SynPETMR-T1-FLAIR | 150 | 2 | 3×10^4^ | 0.2 |  | 2 | 3 | 0.015 |  | 2 | 5 | 0.006 |

Note: For MAPEM-TV, a smoothed TV with smoothing parameter of $\delta=0.01$ was used. The OSL algorithm was then used for optimization. For SENSE-TV reconstructions, an exact TV prior was used. For simulated and real PET-MR data the penalty parameter of the ADMM algorithm was set to $\rho=1$, while for the in-vivo MR data it was set to $\rho=0.1$. See (3) for more details of both the OSL and ADMM algorithms.


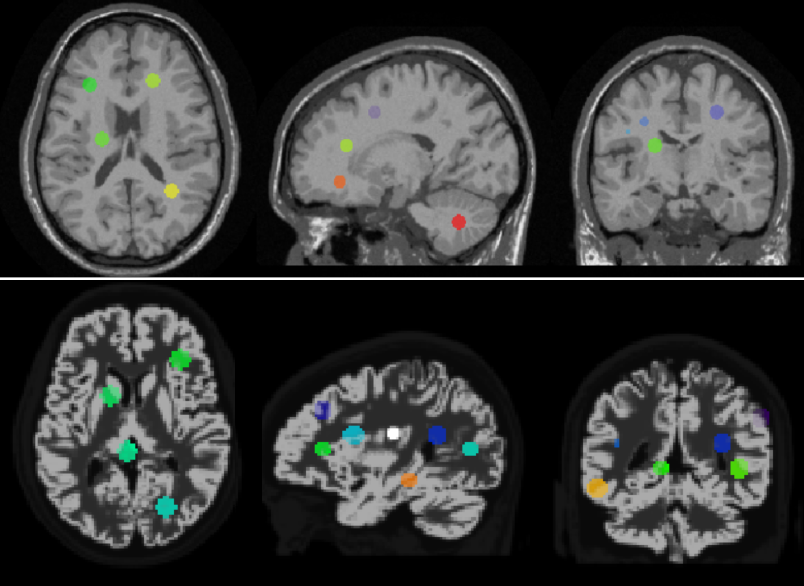


**Supporting Information Figure S1**. Background ROIs used for calculation of CNR in the simulated brain phantom.


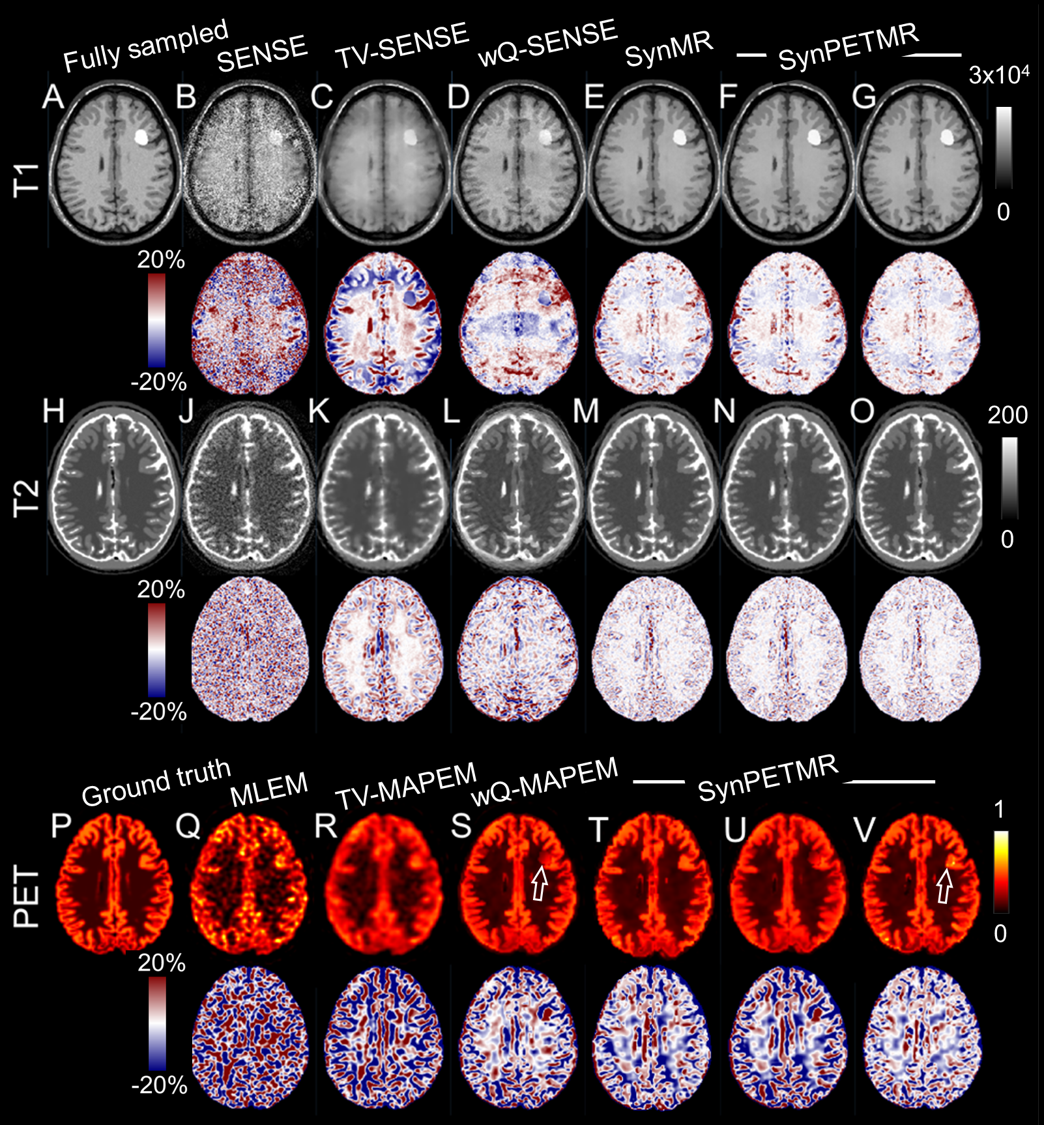


**Supporting Information Figure S2**. Same as Figure 1, but with added voxel-wise error maps.

**
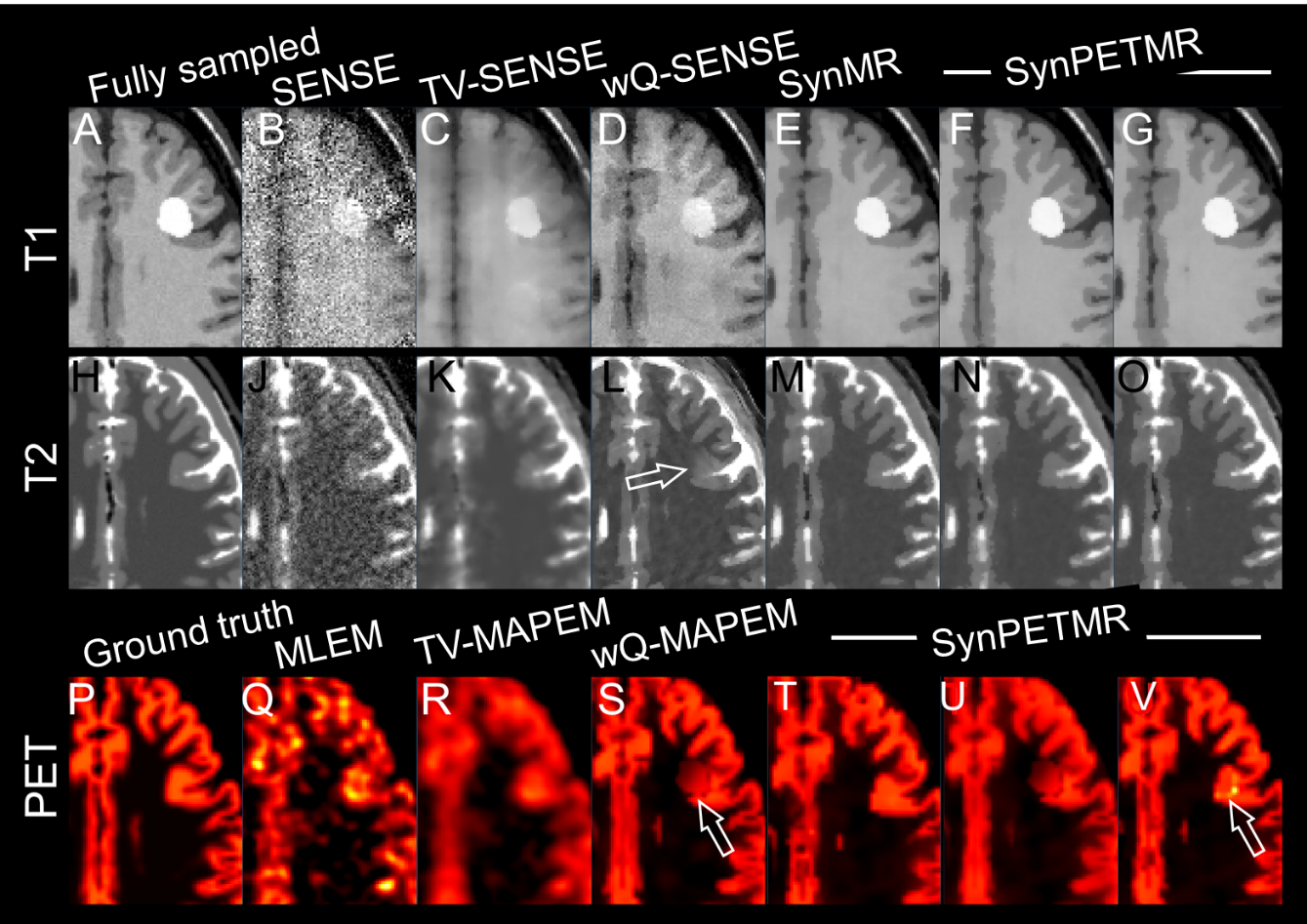
**

**Supporting Information Figure S3**. Zoomed-in of Figure 1.


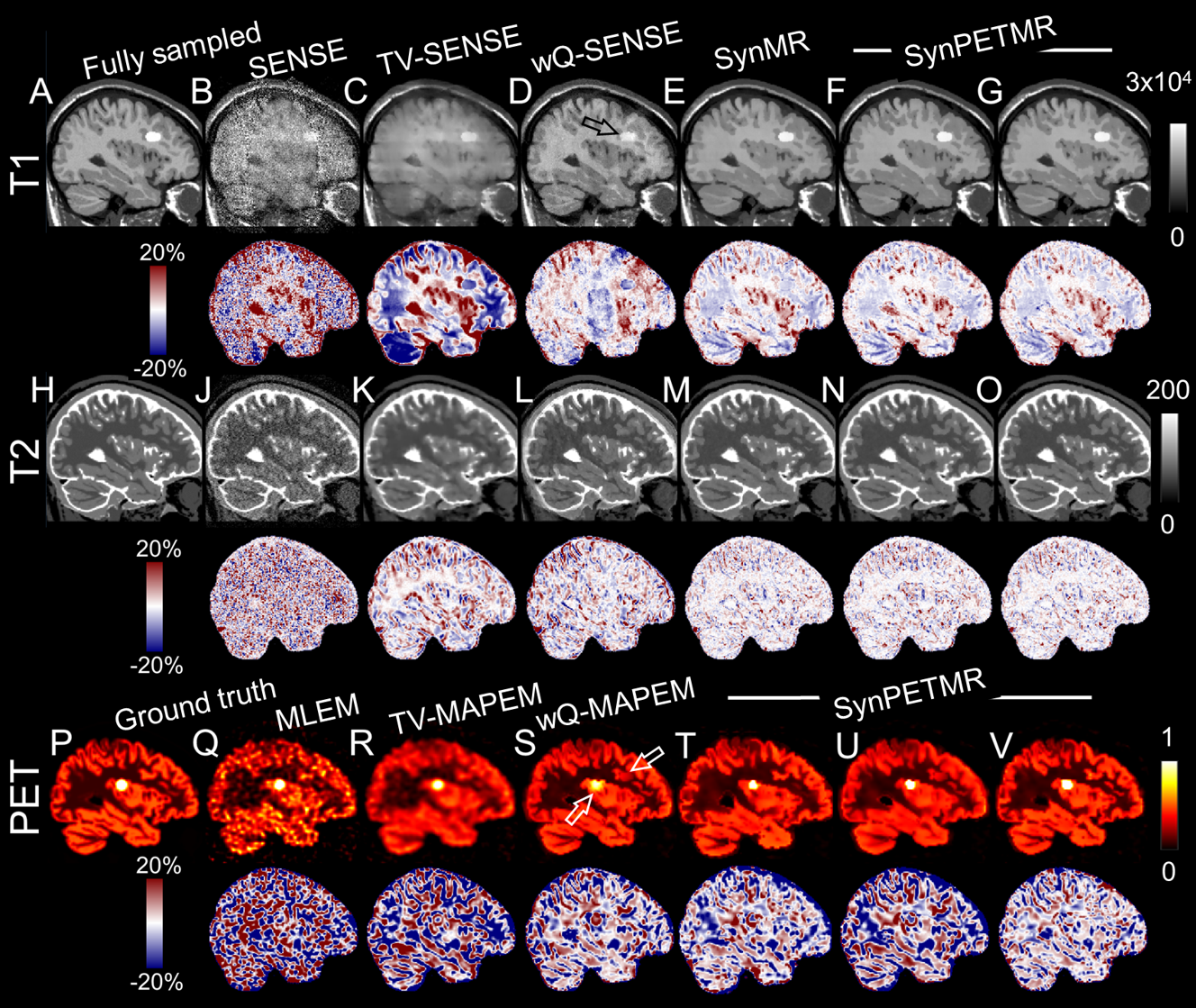


**Supporting Information Figure S4**. Same as Figure 2, but with added voxel-wise error maps.


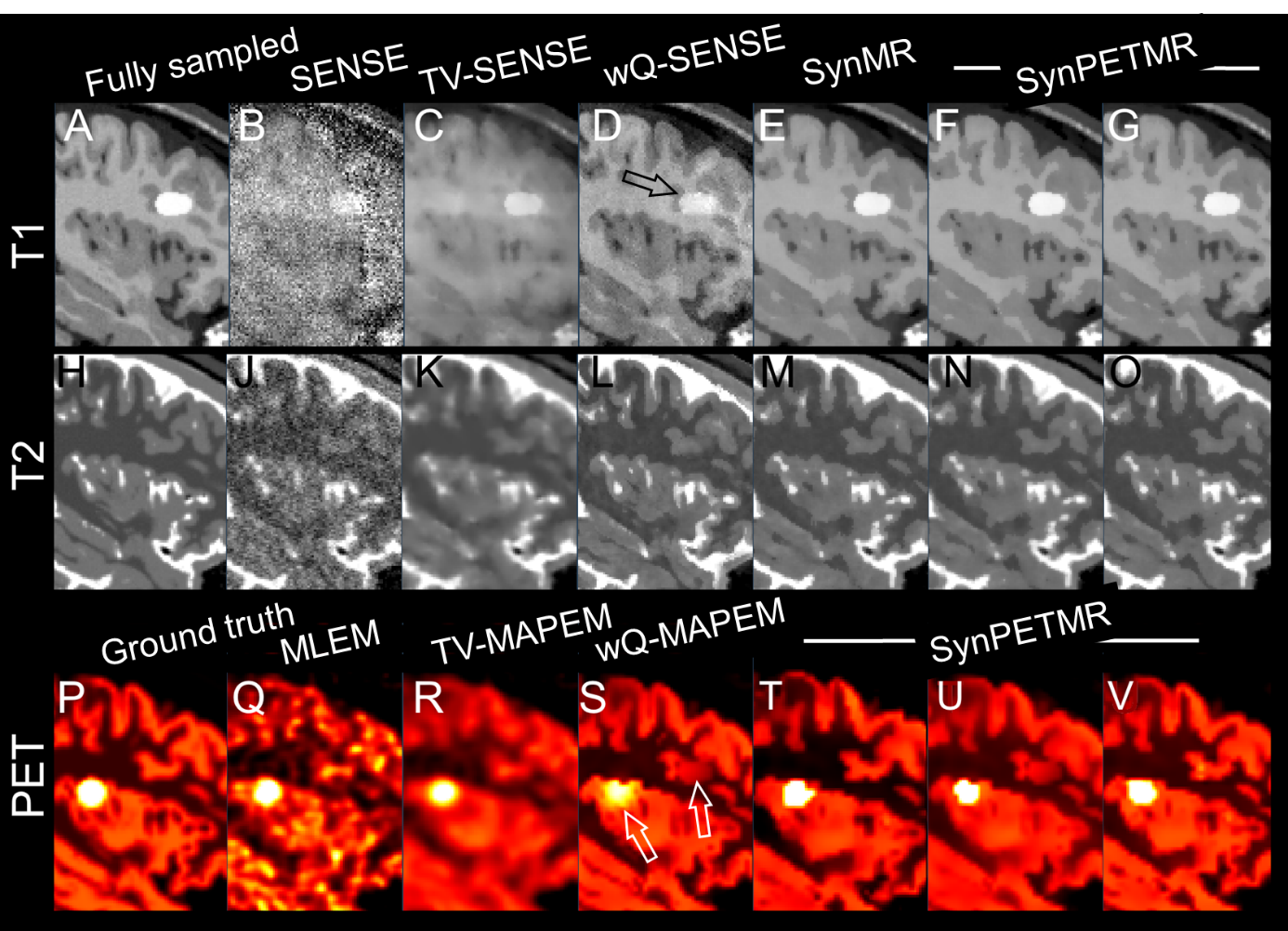


**Supporting Information Figure S5**. Zoomed-in of Figure 2.

**Supporting Information Figure S6**. Convergence of the reconstruction methods in terms of normalized root mean square error (NRMSE) in the whole brain for each image update of the simulated PET, T1 and T2 MR datasets.


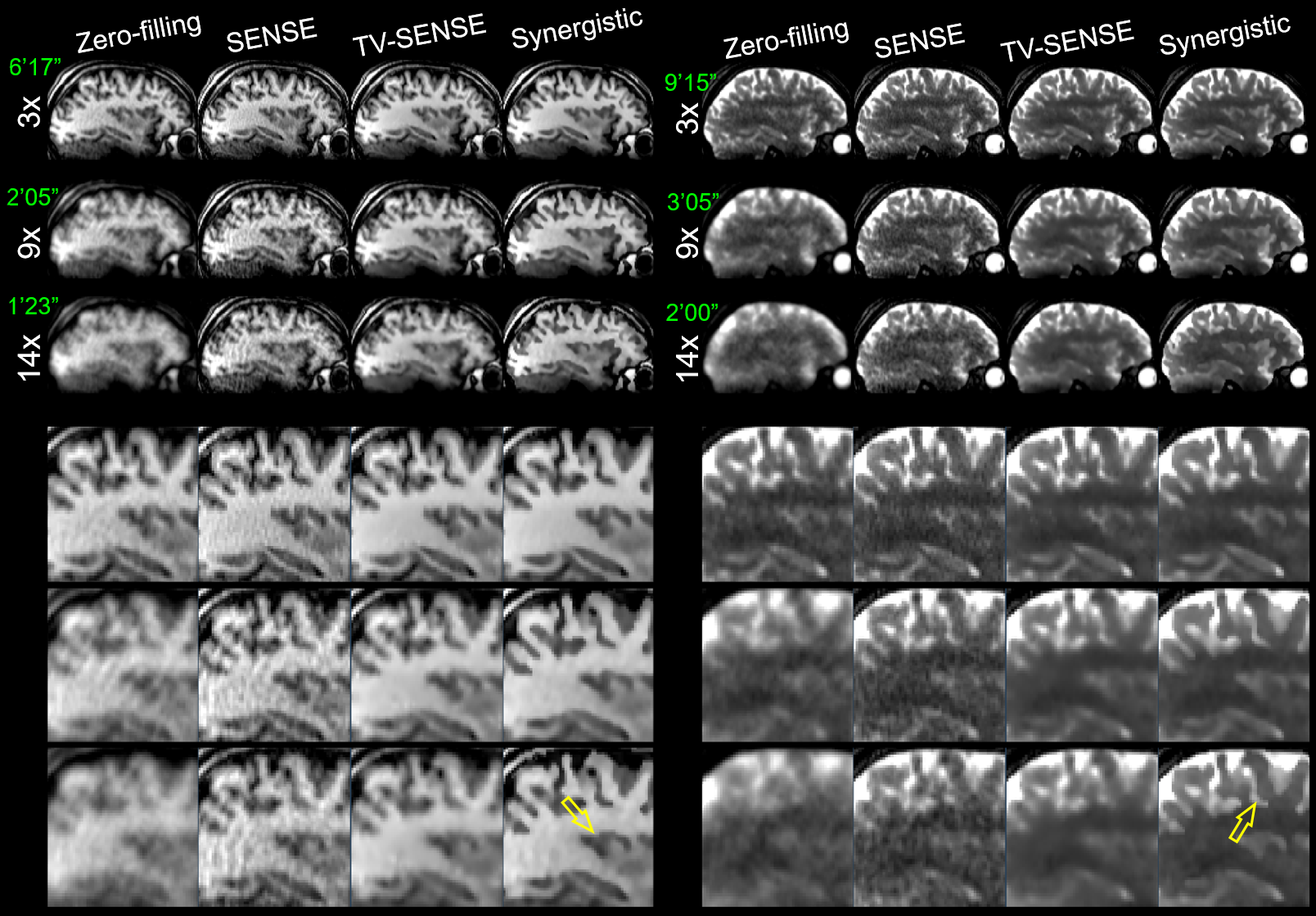


**Supporting Information Figure S7.** Synergistic reconstruction of the prospectively undersampled T1 (left) and T2 (right) datasets for the first healthy volunteer. The acceleration factor and resulting acquisition time (in minutes and seconds) of each scan are shown.


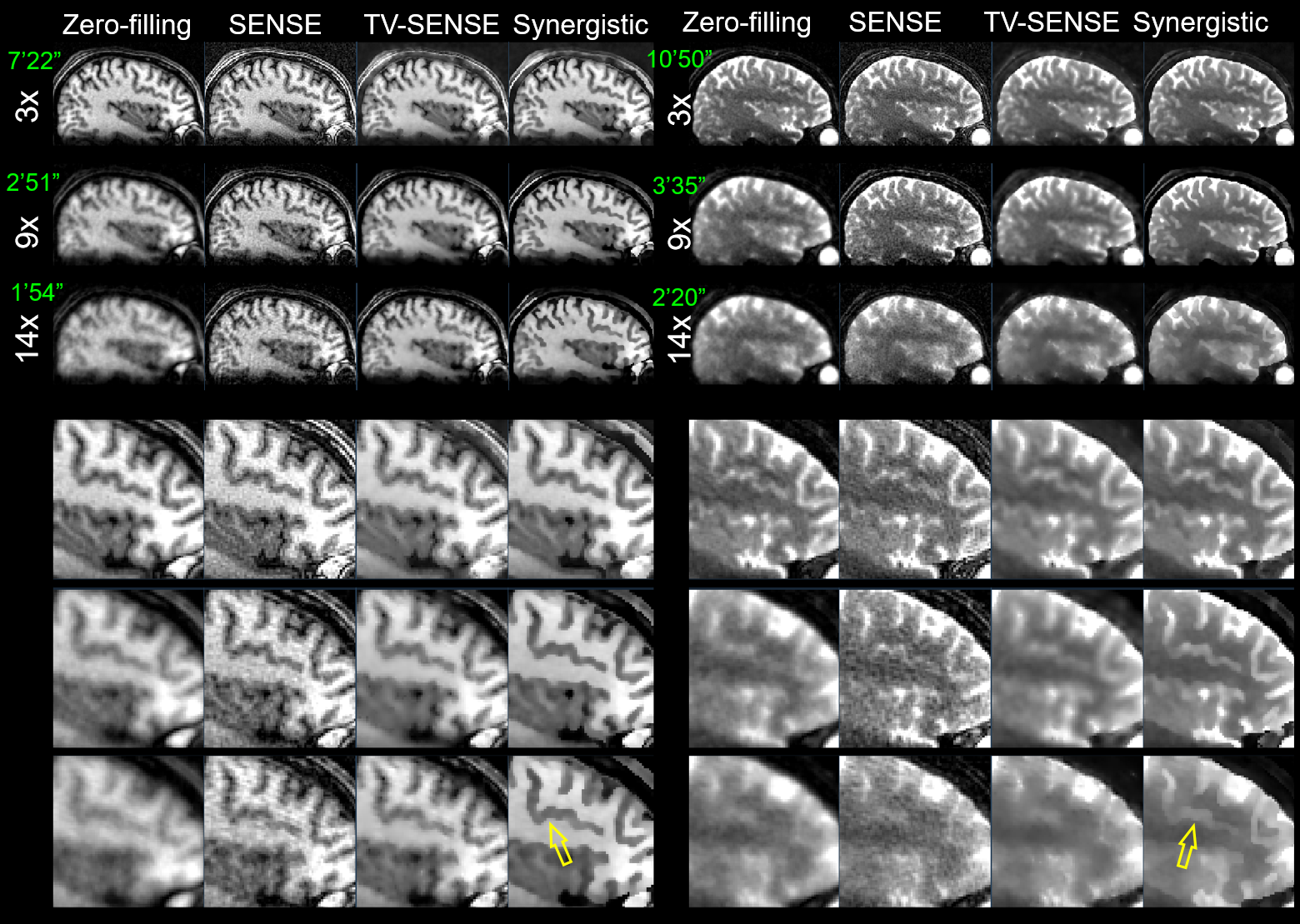


**Supporting Information Figure S8.** Synergistic reconstruction of the prospectively undersampled T1 (left) and T2 (right) datasets for the second healthy volunteer. Acceleration factor and resulting acquisition time (in minutes and seconds) of each scan are shown.

| Volunteer #1 | 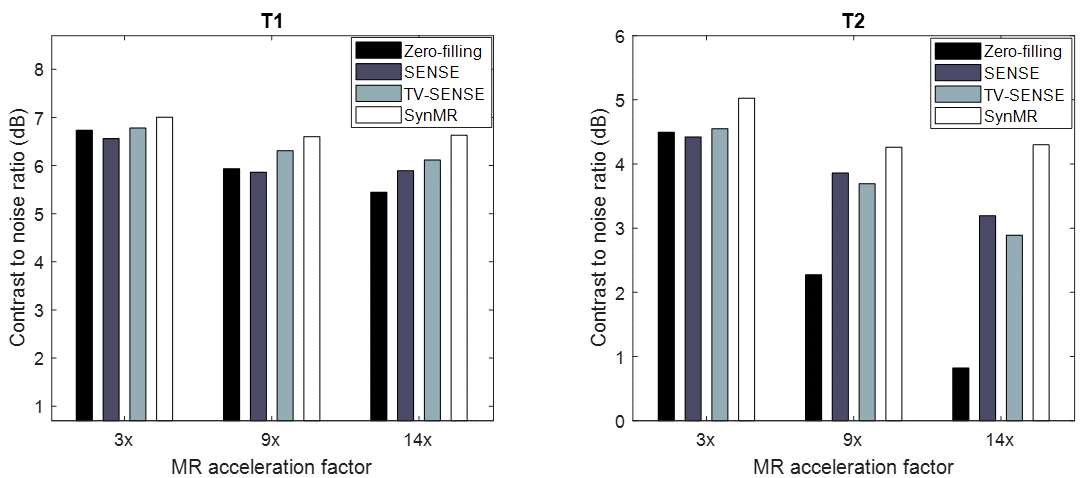 |
| --- | --- |
| Volunteer #2 | 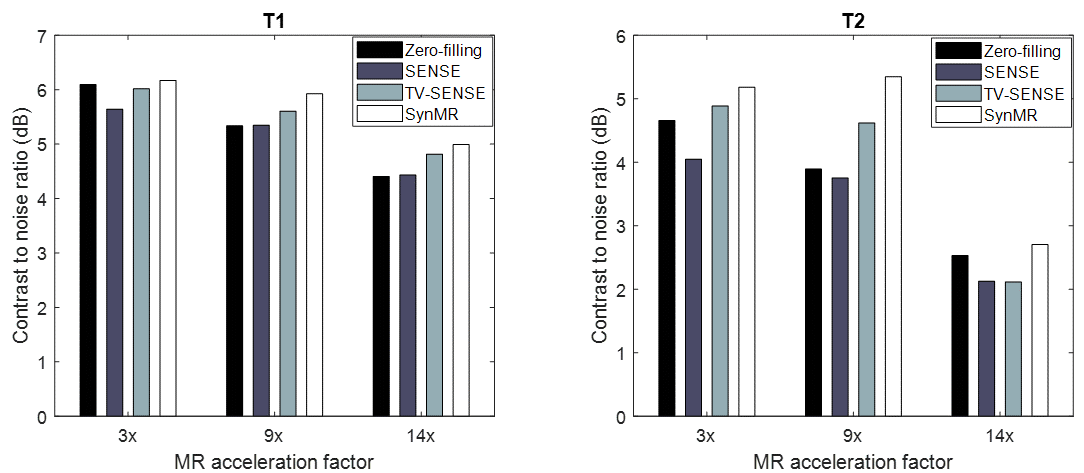 |

**Supporting Information Figure S9**. CNR between grey and white matter of the T1 and T2 images of the in-vivo MR datasets.

**
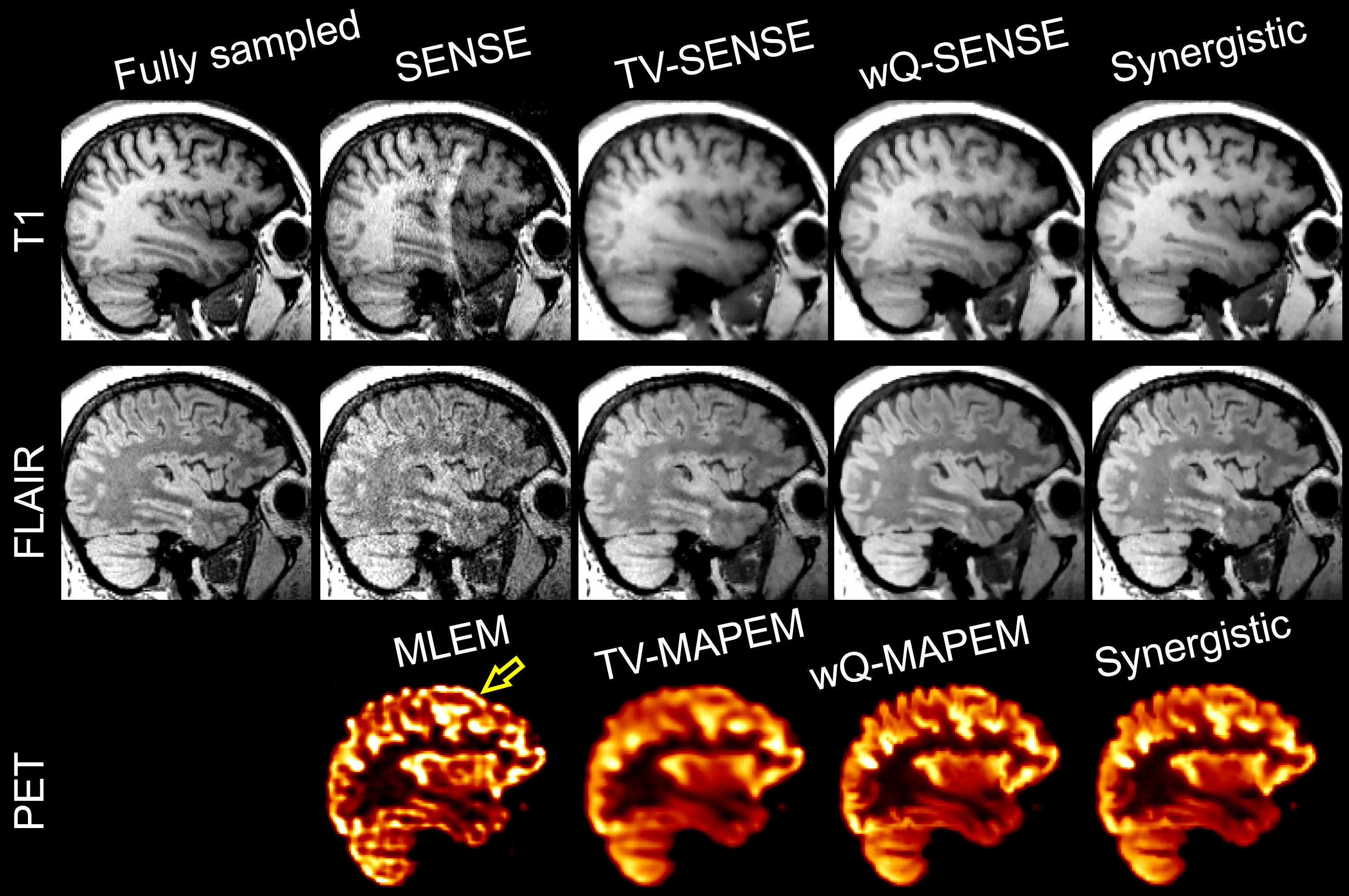
**

**Supporting Information Figure S10**. Same as Figure 9 but for a sagittal slice. The arrow indicates Gibbs artefacts in the PET MLEM reconstruction.


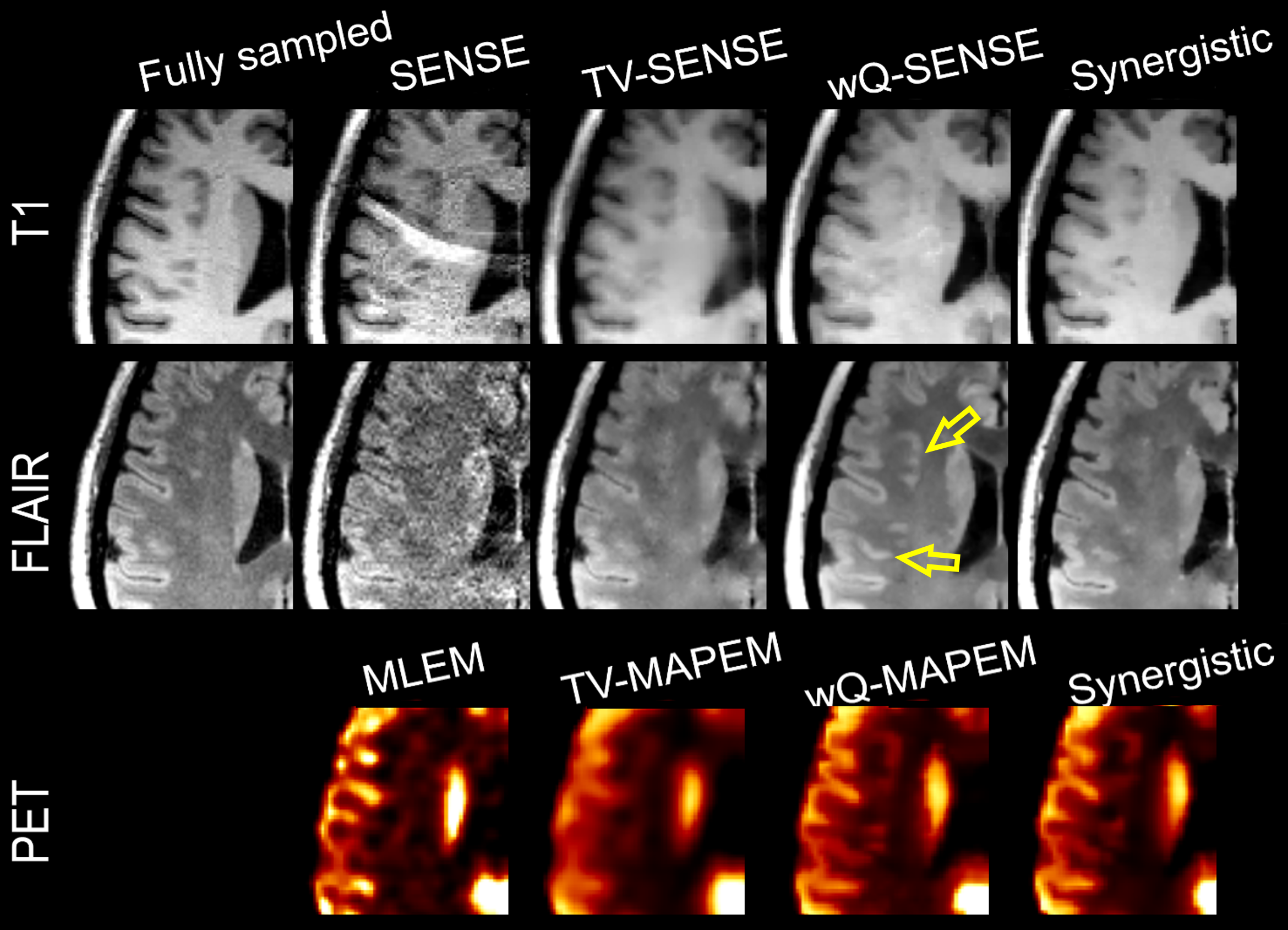


**Supporting Information Figure S11**. Zoomed in from Figure 9. The arrows point to structural artefacts induced by T1-guidance of the FLAIR reconstruction, (i.e. wQ-SENSE).

**Supporting Information Figure S12.** CNR between grey and white matter of the FDG-PET, T1 and FLAIR images of the in-vivo PET-MR dataset.


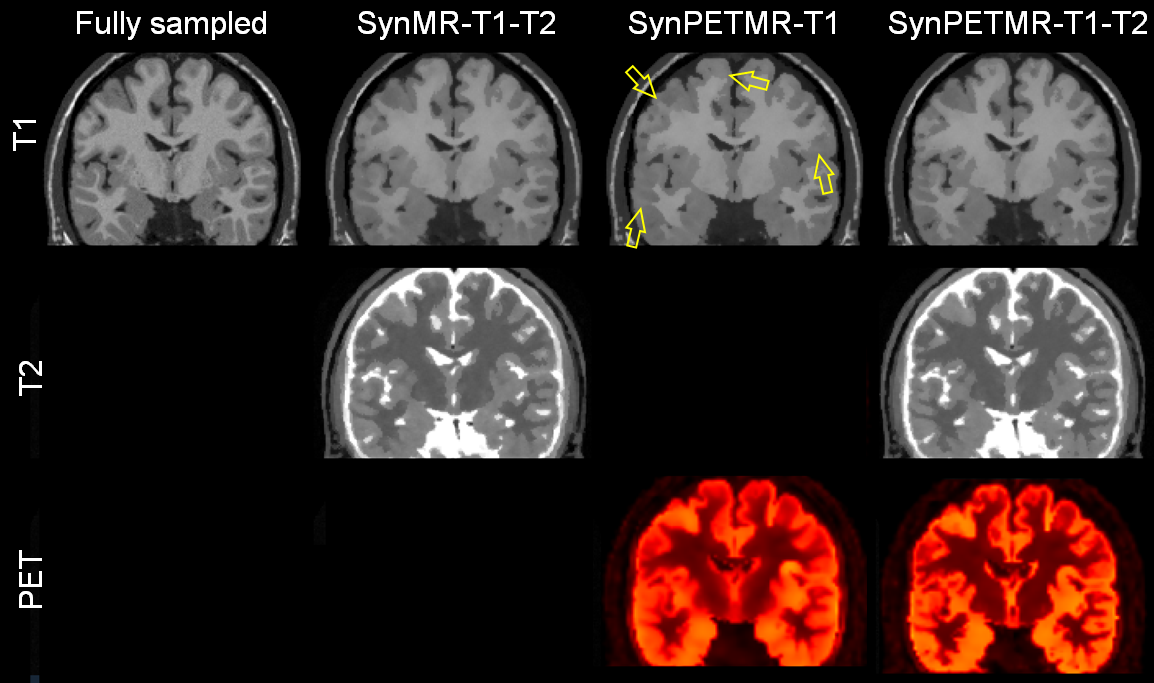


**Supporting Information Figure S13**. Comparison of different synergistic reconstructions of the simulated PET-MR dataset for synergistic reconstruction of T1 and T2 (SynMR-T1-T2), PET and T1 (SynPETMR-T1), and PET, T1 and T2 (SynPETMR-T1-T2). Comparison of SynMR-T1-T2 and SynPET-MR-T1 shows that the T1 image has been improved more when synergistically reconstructed with the T2 image than the PET image (see arrows). Synergistic reconstruction of all data together (in SynPETMR-T1-T2) is beneficial for all reconstructions.


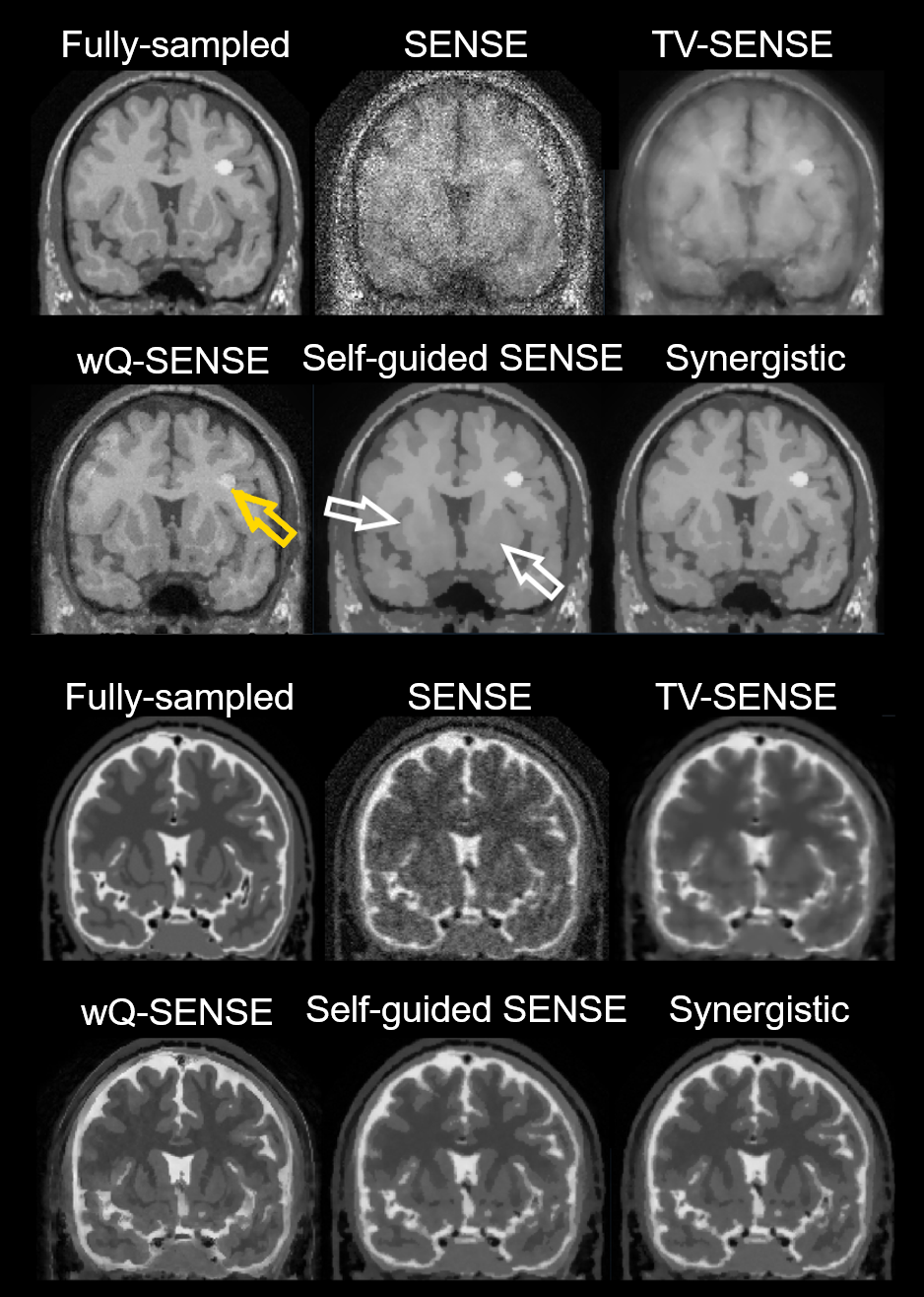


**Supporting Information Figure S14.** Illustration of the coupling effect of common boundary information between T1 and T2 datasets through their synergistic reconstruction. Compared to self-guided reconstruction, the synergistic one is able to recover more structural details and at the same time preserve unique lesions that are otherwise suppressed by wQ-SENSE.


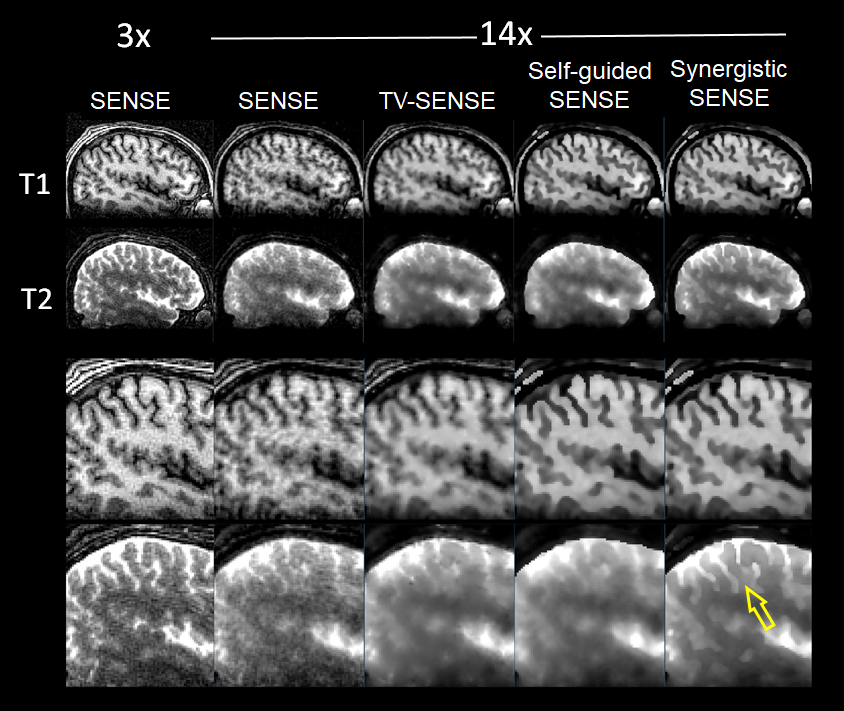


**Supporting Information Figure S15.** Same as Supporting Figure 14, but for the volunteer MR scan #2.


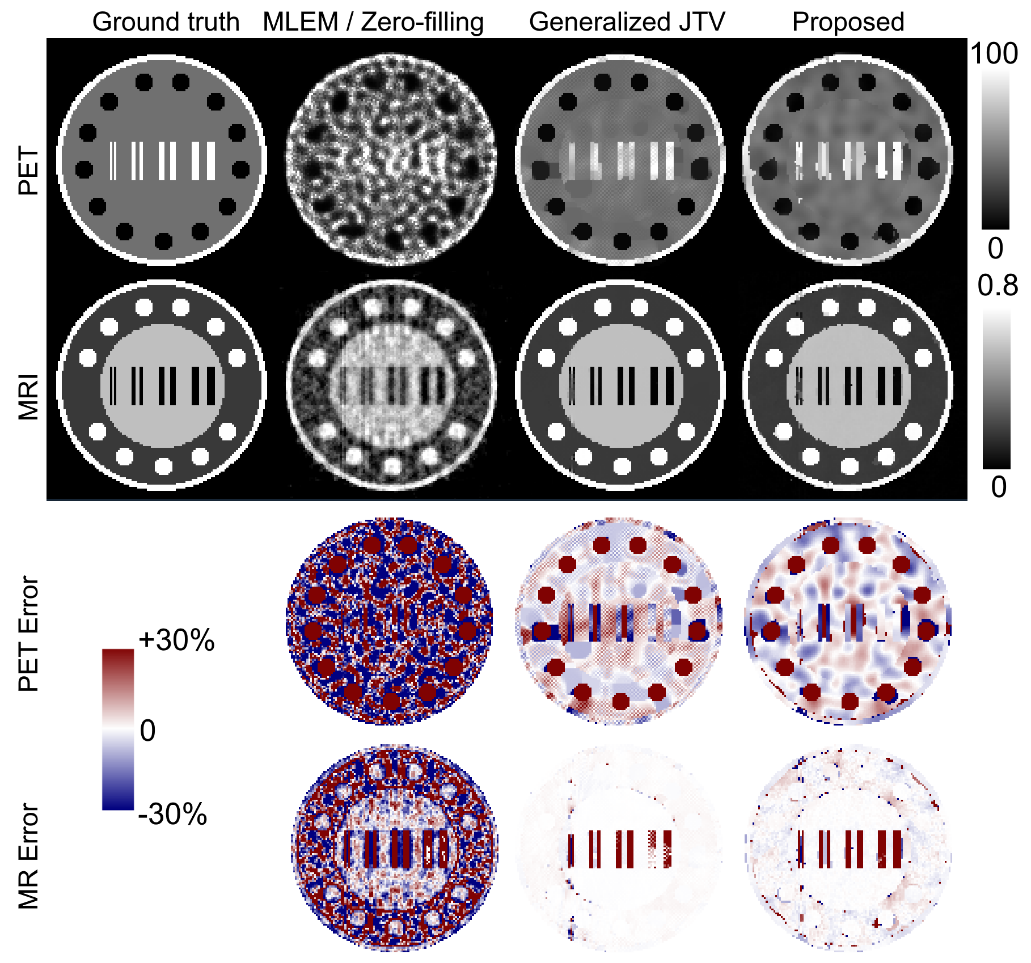


**Supporting Information Figure S16.** Performance comparison of the proposed synergistic reconstruction method with our previous work in (3), using a joint total variation prior generalized using a non-convex potential function, on the resolution phantom proposed by Ehrhardt et al (1) for the ‘*radial 20*’ simulation set-up.


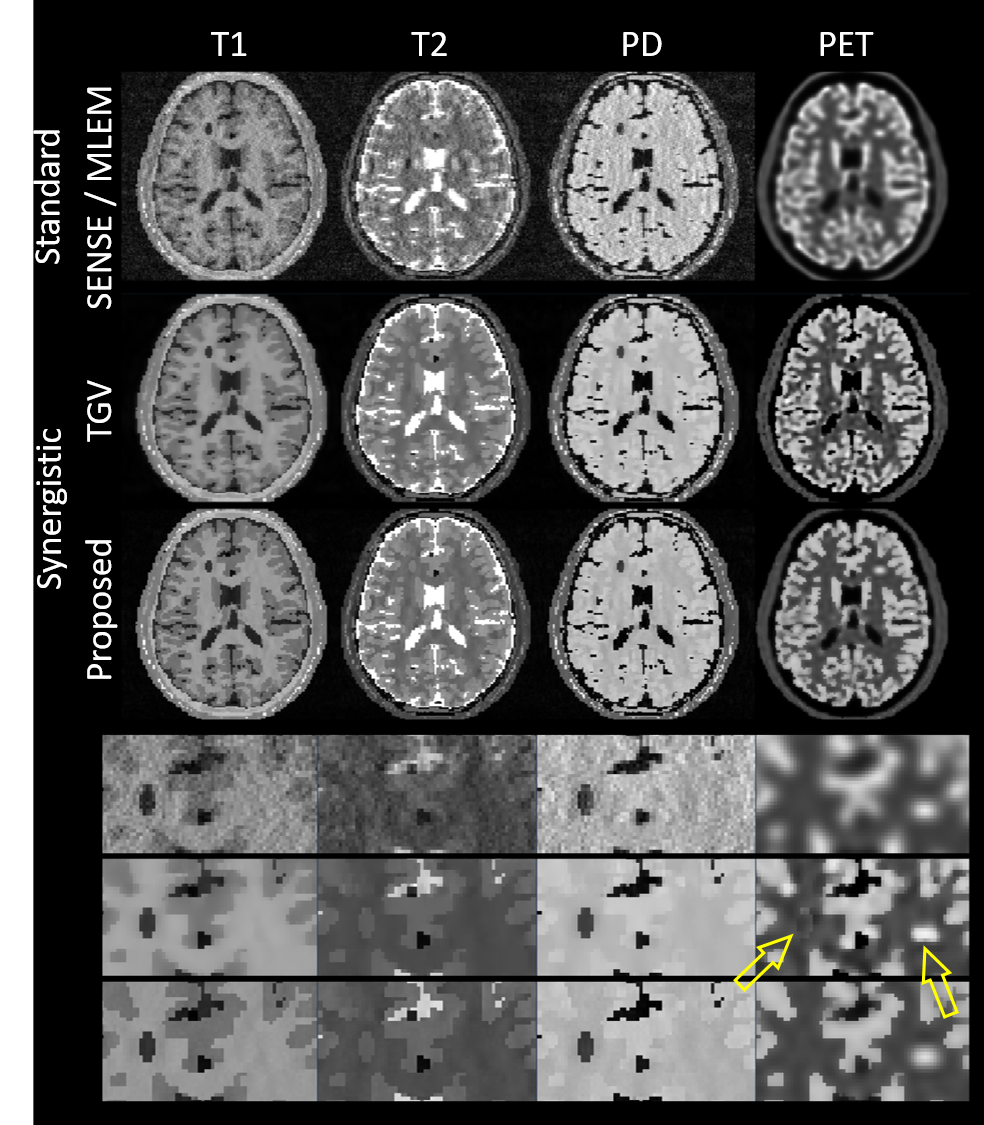


**Supporting Information Figure S17.** Performance comparison of our proposed synergistic algorithm with the synergistic TGV one proposed in Ref (2). In this comparison, the code and simulated dataset were obtained from Ref. (24)
